# Spin-polarized d-orbital filling in cobalt catalysts boosts solution-mediated Li-O_2_ batteries

**DOI:** 10.1093/nsr/nwaf145

**Published:** 2025-04-24

**Authors:** Fengling Zhang, Zhengqiang Hu, Jingning Lai, Nuo Chen, Yuhao Liu, Tianyang Yu, Faiza Arshad, Liyuan Zhao, Nan Chen, Li Li, Qiang Li, Feng Wu, Renjie Chen

**Affiliations:** Beijing Key Laboratory of Environmental Science and Engineering, School of Materials Science and Engineering, Beijing Institute of Technology, Beijing 100081, China; Beijing Key Laboratory of Environmental Science and Engineering, School of Materials Science and Engineering, Beijing Institute of Technology, Beijing 100081, China; Beijing Key Laboratory of Environmental Science and Engineering, School of Materials Science and Engineering, Beijing Institute of Technology, Beijing 100081, China; Beijing Key Laboratory of Environmental Science and Engineering, School of Materials Science and Engineering, Beijing Institute of Technology, Beijing 100081, China; Beijing Key Laboratory of Environmental Science and Engineering, School of Materials Science and Engineering, Beijing Institute of Technology, Beijing 100081, China; Beijing Key Laboratory of Environmental Science and Engineering, School of Materials Science and Engineering, Beijing Institute of Technology, Beijing 100081, China; Beijing Key Laboratory of Environmental Science and Engineering, School of Materials Science and Engineering, Beijing Institute of Technology, Beijing 100081, China; Beijing Key Laboratory of Environmental Science and Engineering, School of Materials Science and Engineering, Beijing Institute of Technology, Beijing 100081, China; Beijing Key Laboratory of Environmental Science and Engineering, School of Materials Science and Engineering, Beijing Institute of Technology, Beijing 100081, China; Innovative Research Team in High-Safety Energy Storage System and Smart Microgrids of Guangdong Province, Beijing Institute of Technology (Zhuhai), Zhuhai 519088, China; Shandong Key Laboratory of Advanced Chemical Energy Storage and Intelligent Safety, Advanced Technology Research Institute, Beijing Institute of Technology, Jinan 250300, China; Beijing Key Laboratory of Environmental Science and Engineering, School of Materials Science and Engineering, Beijing Institute of Technology, Beijing 100081, China; Innovative Research Team in High-Safety Energy Storage System and Smart Microgrids of Guangdong Province, Beijing Institute of Technology (Zhuhai), Zhuhai 519088, China; Shandong Key Laboratory of Advanced Chemical Energy Storage and Intelligent Safety, Advanced Technology Research Institute, Beijing Institute of Technology, Jinan 250300, China; Collaborative Innovation Center of Electric Vehicles in Beijing, Beijing 100081, China; College of Physics, Weihai Innovation Research Institute, Institute of Materials for Energy and Environment, Qingdao University, Qingdao 266071, China; Beijing Key Laboratory of Environmental Science and Engineering, School of Materials Science and Engineering, Beijing Institute of Technology, Beijing 100081, China; Innovative Research Team in High-Safety Energy Storage System and Smart Microgrids of Guangdong Province, Beijing Institute of Technology (Zhuhai), Zhuhai 519088, China; Shandong Key Laboratory of Advanced Chemical Energy Storage and Intelligent Safety, Advanced Technology Research Institute, Beijing Institute of Technology, Jinan 250300, China; Collaborative Innovation Center of Electric Vehicles in Beijing, Beijing 100081, China; Beijing Key Laboratory of Environmental Science and Engineering, School of Materials Science and Engineering, Beijing Institute of Technology, Beijing 100081, China; Innovative Research Team in High-Safety Energy Storage System and Smart Microgrids of Guangdong Province, Beijing Institute of Technology (Zhuhai), Zhuhai 519088, China; Shandong Key Laboratory of Advanced Chemical Energy Storage and Intelligent Safety, Advanced Technology Research Institute, Beijing Institute of Technology, Jinan 250300, China; Collaborative Innovation Center of Electric Vehicles in Beijing, Beijing 100081, China

**Keywords:** sluggish reaction kinetics, nucleation and decomposition, catalyst, spin-polarized, solution-mediated pathway

## Abstract

The sluggish reaction kinetics in current Li-O_2_ batteries (LOBs) hinder the efficient nucleation and decomposition of insulating Li_2_O_2_ on catalyst surfaces. Therefore, developing effective strategies to regulate Li_2_O_2_ growth and elucidating the catalytic mechanism are essential for unlocking the full potential of LOB technology. Herein, a spin-polarized Co-based catalyst exhibits a significant reversible magnetization change (9.5 emu g^−1^) during the oxygen reduction and evolution reactions. The strong overlap between the Co 3d and O 2p orbitals modulates the Co 3d orbital occupancy, enhancing spin-electron filling and thereby facilitating rapid O_2_ adsorption and an efficient two-electron transfer process in the initial oxygen reduction reaction step. This optimized electronic structure also promotes the desorption of LiO_2_ intermediates, guiding their disproportionation reactions and enabling Li_2_O_2_ growth via a solution-mediated pathway. Furthermore, the spin-flip effect induced by the internal magnetic field suppresses singlet oxygen (^1^O_2_) formation, effectively mitigating side reactions. As a result, the LOBs demonstrate a remarkably high specific capacity (18 429.6 mAh g^−1^), excellent rate performance and enhanced cycling stability. These findings offer valuable insights into Li_2_O_2_ nucleation mechanisms on high-performance catalysts and provide new design principles for next-generation LOB technologies.

## Teaser

This study modulates the Co 3d orbital occupation of Co-based catalysts, enabling fast O_2_ adsorption, efficient two-electron transfer in the initial reduction step, and solution-phase Li_2_O_2_ growth with suppressed ^1^O_2_ formation.

## INTRODUCTION

Li-O_2_ batteries (LOBs) are receiving interest as a promising technology in the field of secondary batteries, primarily due to their remarkable theoretical specific energy density (3500 Wh kg^−1^) [1–3]. However, current aprotic LOBs encounter a severe performance barrier due to sluggish oxygen reduction and evolution reaction (ORR/OER) kinetics and complex charge transfer mechanisms at the gas–liquid–solid interface. These challenges are primarily associated with the nucleation and decomposition of Li_2_O_2_ on the porous oxygen electrode, where uncontrolled deposition can severely impede charge transport and cycle stability [4,5].

The formation of Li_2_O_2_ follows two distinct pathways: the solution growth pathway and the surface growth pathway [6,7]. In the surface growth pathway, Li_2_O_2_ forms a dense, insulating layer on the oxygen electrode, passivating the electrode surface and increasing polarization, which ultimately leads to rapid capacity degradation [4]. In contrast, Li_2_O_2_ formed via the solution growth pathway remains well dispersed in the electrolyte, reducing electrode passivation and enabling higher discharge capacities. To promote the solution growth mechanism and enhance LOB performance, several strategies have been explored, including high donor-number electrolytes to increase Li⁺ solvation, [8–10] functional additives to regulate solvation structure, [11] and redox mediators (RMs) to facilitate charge transfer [1,12]. However, these approaches focus on improving LiO_2_ solubility and its disproportionation into Li_2_O_2_. The initial step—the selective formation of LiO_2_ (O_2 _+ e^−^ + Li^+^ → LiO_2_) before its further reduction—remains relatively underexplored. This rate-determining step (RDS) is strongly influenced by the catalytic properties of the air electrode, [13] as it dictates whether LiO_2_ remains in solution or undergoes excessive electron transfer to form Li_2_O_2_ prematurely.

The nucleation pathway of Li_2_O_2_ is governed by the following key reactions: solution growth pathway (reactions (1) and (2)) and surface growth pathway (reactions (3) and (4)):


(1) \begin{eqnarray*}
{\mathrm{2}}{{\mathrm{O}}_{\mathrm{2}}} + {\mathrm{2}}{{\mathrm{e}}^{\mathrm{ -}}} + {\mathrm{2L}}{{\mathrm{i}}^{\mathrm{ + }}} \to {\mathrm{2Li}}{{\mathrm{O}}_{\mathrm{2}}},
\end{eqnarray*}

(2) \begin{eqnarray*}
{\mathrm{2Li}}{{\mathrm{O}}_{\mathrm{2}}} \to {\mathrm{L}}{{\mathrm{i}}_{\mathrm{2}}}{{\mathrm{O}}_{\mathrm{2}}} + {{\mathrm{O}}_{\mathrm{2}}},
\end{eqnarray*}

(3) \begin{eqnarray*}
{{\mathrm{O}}_{\mathrm{2}}} + {{\mathrm{e}}^{\mathrm{ - }}} + {\mathrm{L}}{{\mathrm{i}}^{\mathrm{ + }}} \to {\mathrm{Li}}{{\mathrm{O}}_{\mathrm{2}}},
\end{eqnarray*}

(4) \begin{eqnarray*}
{\mathrm{Li}}{{\mathrm{O}}_{\mathrm{2}}} + {{\mathrm{e}}^{\mathrm{ - }}} + {\mathrm{ L}}{{\mathrm{i}}^{\mathrm{ + }}} \to {\mathrm{2L}}{{\mathrm{i}}_{\mathrm{2}}}{{\mathrm{O}}_{\mathrm{2}}}.
\end{eqnarray*}

In the solution pathway, the electron transfer predominantly occurs in the first step (reaction (1)), ensuring that LiO_2_ remains in solution and can disproportionate to Li_2_O_2_ via reaction (2). Conversely, in the surface pathway, LiO_2_ undergoes a second electron transfer (reaction (4)), leading to Li_2_O_2_ nucleation on the oxygen electrode surface. Therefore, enhancing the first-step electron transfer efficiency while suppressing further reduction of LiO_2_ is crucial for optimizing the LOB reaction mechanism.

Spin-dependent electron transfer plays a pivotal role in governing this process. The ORR/OER kinetics in LOBs involve intrinsic spin selection rules, where the interaction between transition metal (TM) d orbitals and O_2_ molecular orbitals influences charge transfer efficiency [14,15]. TM catalysts with ferromagnetic (FM) ordering generate internal magnetic fields that induce spin polarization and spin-flip effects, which can modulate electron distribution at the catalyst surface [16,17]. Specifically, the degree of d orbital filling and its overlap with O 2p orbitals determine the catalytic activity and selectivity for LiO_2_ formation. By tuning the d-band center, the adsorption properties of catalyst surface can be adjusted to favor moderate LiO_2_ binding strength, which prevents excessive electrochemical reduction while ensuring sufficient LiO_2_ stability for disproportionation.

**Figure 1.**
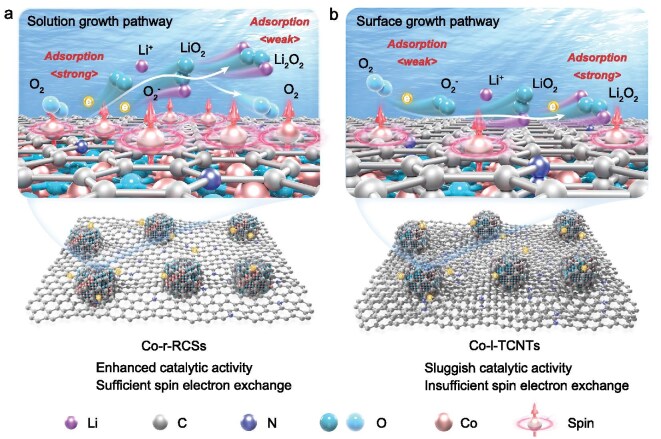
(a) Co-r-RCSs catalyst strongly adsorbs O_2_ for sufficient orbital spin electron exchange to promote selective electron transfer and stabilize LiO_2_/Li_2_O_2_ in solution. (b) Co-l-TCNTs catalyst weakly adsorbs O_2_ for insufficient orbital spin electron exchange to guide the formation of Li_2_O_2_ with surface growth pathway.

Herein, we report a ferromagnetically ordered Co-based catalyst with optimized d-orbital spin states to selectively regulate Li_2_O_2_ growth in LOBs. Compared to conventional catalysts, the Co-rich catalyst with reduced carbon shells (Co-r-RCSs) provides more exposed metal sites and a higher electrochemical surface area (ECSA), while the Co-less catalyst with thick carbon nanotubes (Co-l-TCNTs) exhibits lower catalytic activity. In Co-r-RCSs, the strong Co 3d-O 2p orbital overlap effectively shifts the d-band center, enhancing O_2_ adsorption while promoting selective electron transfer to stabilize LiO_2_ in solution rather than driving its further electrochemical reduction (Fig. 1). Magnetic characterization confirms a reversible magnetization change of 9.5 emu g^−1^ during ORR/OER, as opposed to only 4.4 emu g^−1^ in Co-l-TCNTs, indicating robust spin-electron exchange dynamics. The weak LiO_2_ binding interaction on Co-r-RCSs further facilitates the solution-mediated Li_2_O_2_ growth mechanism, while the internal magnetic-field-induced spin-flip effect suppresses singlet oxygen (^1^O_2_) formation, minimizing parasitic side reactions. Notably, LOBs employing Co-r-RCSs achieve an ultra-high discharge capacity (18 429.6 mAh g^−1^), low overpotential (0.75 V), enhanced rate performance and prolonged cycling stability (240 cycles at 1000 mAh g^−1^). This study provides a systematic framework for understanding spin-dependent Li_2_O_2_ nucleation on high-performance catalysts, thereby inspiring new possibilities for the development of LOBs.

## RESULTS AND DISCUSSION

### Physicochemical characterizations of catalysts

**Figure 2.**
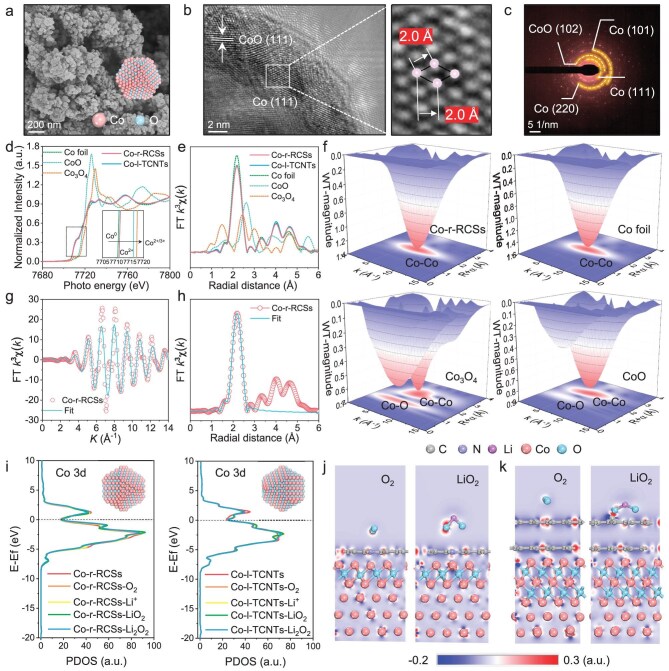
(a and b) SEM and HRTEM images and corresponding enlarged figures and (c) SAED patterns of Co-r-RCSs. (d and e) Co K-edge XANES spectra and FT-EXAFS fitting curves at K-edge in R-space for the Co-r-RCSs, Co-l-TCNTs, Co foil, CoO and Co_3_O_4_. (f) WT-EXAFS of Co-r-RCSs, Co foil, CoO and Co_3_O_4_. (g and h) Fit of FT-EXAFS spectra of Co-r-RCSs at k space and R space. (i) Computed PDOS of Co-r-RCSs and Co-l-TCNTs before and after adsorption of different species. Charge density difference plots of different adsorbates on (111) surface for (j) Co-r-RCSs and (k) Co-l-TCNTs.

To precisely tailor the pore structure, electronic properties and adsorption behavior of catalysts with reactive oxygen species (ROS), we employed a carbothermal reduction strategy at varying temperatures to process the Co-based precursor, resulting in catalysts with distinct compositions and structures (photographs of the different samples are shown in Fig. S1). The scanning electron microscope (SEM) images of the precursor material and the products treated at 700°C are presented in Figs S2 and S3. These images reveal that the annealed sample exhibits a morphology characterized by metal nanoparticles encapsulated within thick carbon nanotubes (Co-l-TCNTs). It should be explained that in the absence of externally introduced CNT precursors during synthesis, the calcined Co metal in an inert atmosphere facilitates the *in-situ* formation of CNTs through the ‘tip-growth’ mechanism [18,19]. Upon annealing the precursor at 900°C, the catalyst morphology transforms into metal nanoparticles enclosed within reduced carbon shells rather than CNTs (Co-r-RCSs, Fig. 2a). SEM imaging of the sample treated at an elevated temperature of 1100°C reveals a compact, block-like morphology (Fig. S4), which is mechanically robust and challenging to grind. Consequently, Co-l-TCNTs and Co-r-RCSs were selected for further investigation. To gain deeper structural insights, we employed transmission electron microscopy (TEM) to examine the composition and lattice arrangement of the catalysts. X-ray energy dispersive spectroscopic (EDS) mapping demonstrates the uniform distribution of Co, C, O and N elements in both Co-r-RCSs and Co-l-TCNTs samples (Figs S5 and S6, and Tables S1 and S2). High-resolution TEM (HRTEM) and selected area electron diffraction (SAED) analyses confirm that Co-r-RCSs predominantly consist of Co and CoO phases (Fig. 2b and c), exhibiting well-defined lattice fringes with spacings of 0.245 nm and 0.204 nm, corresponding to the CoO (111) and Co (111) planes, respectively. Similarly, the Co-l-TCNT structure also incorporates the characteristic Co (111) and CoO (200) lattice planes (Fig. S7).

To further confirm the specific valence state of Co species, the X-ray absorption near-edge structure (XANES) spectra of Co K-edge demonstrate that both the intensity and position of the fingerprint peak for Co-r-RCSs and Co-l-TCNTs are positioned near the standard Co foil, with a slight shift toward CoO (Fig. 2d). This indicates that the Co species are predominantly 0 valence state, with a partial presence of +2 oxidation state [20]. The Fourier transform k3-weighted extended X-ray absorption fine structure (FT-EXAFS) spectra, as shown in Fig. 2e (R space) and Fig. S8 (k space), reveal a distinct shell at 2.17 Å for both samples, corresponding precisely to the Co-Co bond distance (2.17 Å) observed in Co foil. Moreover, wavelet transform analysis of the EXAFS (WT-EXAFS) signal shows a peak at 6.4 Å^−1^, which is assigned to the Co-Co contribution (Fig. 2f and Fig. S9). The fitted curve in k space (Fig. 2g) also aligns well with the experimental data. The Co K-edge EXAFS fitting for the first shell in R space (Fig. 2h and Fig. S10) confirms the presence of the Co-Co bond. Further X-ray diffraction (XRD) characterization confirms that both samples consist of Co and CoO (Fig. S11). To distinguish the composition ratio of the two catalysts, thermogravimetry (TG) tests were conducted under air and Ar/H_2_ (4% H_2_) atmospheres, revealing that the Co composition ratio of Co-r-RCSs (Co: 80.15%, CoO: 17.25%, C: 2.6%) is significantly higher than that of Co-l-TCNTs (Co: 65.56%, CoO: 27.94%, C: 6.5%) (Figs S12 and S13). This indicates that the higher-temperature treatment facilitates the reduction of CoO to Co through *in-situ* carbonization, thereby exposing more metal sites. The magnetic properties of the two samples were further compared using magnetometry. The magnetic hysteresis (MH) curves (Fig. S14) reveal that the saturation magnetization (M_S_) of Co-r-RCSs (108.4 emu g^−1^) is higher than that of Co-l-TCNTs (98.6 emu g^−1^), further validating the increased Co content and improved electrical conductivity. Meanwhile, Raman characterization was used to analyze vibrational modes, which indirectly reflect the molecular orbital interactions of the samples. Fig. S15 shows distinct characteristic peaks at 456, 503, 595 and 659 cm^−1^, corresponding to the vibration modes of Co metal/oxide phase [21–23]. The Raman spectrum of Co-r-RCSs shows that the D and G peaks of C at 1350 and 1580 cm^−1^ disappear, indicating that the carbon coating in the Co-r-RCSs sample was reduced. This structural difference on the surface significantly influence the catalytic activity, and consequently result in distinct ORR/OER kinetics [24]. X-ray photoelectron spectroscopy (XPS) spectra (Figs S16 and S17) illustrate that the valence states of the Co species in both Co-r-RCSs and Co-l-TCNTs samples include Co^0^ (778.3 eV) and Co^2+^ (780.2 eV) [25]. Significantly, the Co-r-RCSs sample displays a higher proportion of Co^0^ and lower proportion of N and Co-O, aligning with the results from TG and magnetic tests.

Electronic density of states (DOS) calculations for Co-r-RCSs and Co-l-TCNTs were performed to investigate the adsorption behavior of ROS on the catalysts. Figure 2i and Fig. S20 show the calculated partial DOS (PDOS) for Co-r-RCSs and Co-l-TCNTs before and after the adsorption of different Li^+^ and O_2_-related species. The Co 3d and O 2p centers are determined for Co-r-RCSs and Co-l-TCNTs by integrating the PDOS. Our findings (Tables S3 and S4) indicate that the Co 3d and O 2p centers are closer in proximity in the Co-r-RCSs catalyst. This increased Co-O covalency facilitates electron transfer between the TM cation and O_2_ adsorbates, promoting electron injection/extraction, and ultimately accelerating the rates of both ORR and OER [26]. More importantly, the relatively low d band center of Co 3d in Co-r-RCSs favors the reduction of the binding energy of the intermediates and catalyst, thus lowering the reaction energy barrier [27]. During the adsorption of different species, the shift in the d band center of the Co-r-RCSs is mainly due to the energy transition between Co 3d and O 2p orbitals. The charge density difference plots and adsorption energy of different intermediates adsorbed on (111) surface for Co-r-RCSs and Co-l-TCNTs are shown in Fig. 2j and k, and Figs S21 and S22. The Co-r-RCSs catalyst exhibits stronger adsorption on O_2_, with larger increased charged areas predominantly observed at Co sites, indicating that Co plays a crucial role in the electron-withdrawing interactions with the intermediate species. Additionally, the adsorption energy and corresponding optimized structure of different intermediates on the electrodes are calculated, as shown in Figs S23–S25. The adsorption energies of Co-r-RCSs for O_2_, LiO_2_ and Li_2_O_2_ are −2.41, −1.64 and −1.86 eV, respectively, compared with those of Co-l-TCNTs, with values of −1.91, −5.61 and 2.51 eV, respectively. The weak adsorption of Co-r-RCSs toward LiO_2_ and Li_2_O_2_ facilitates their desorption from the electrode surface, directly guiding the solution growth pathway, which also supports the decomposition of Li_2_O_2_ in the OER process, mitigating electrode deactivation [28].

### Electrochemical performance of LOBs

**Figure 3.**
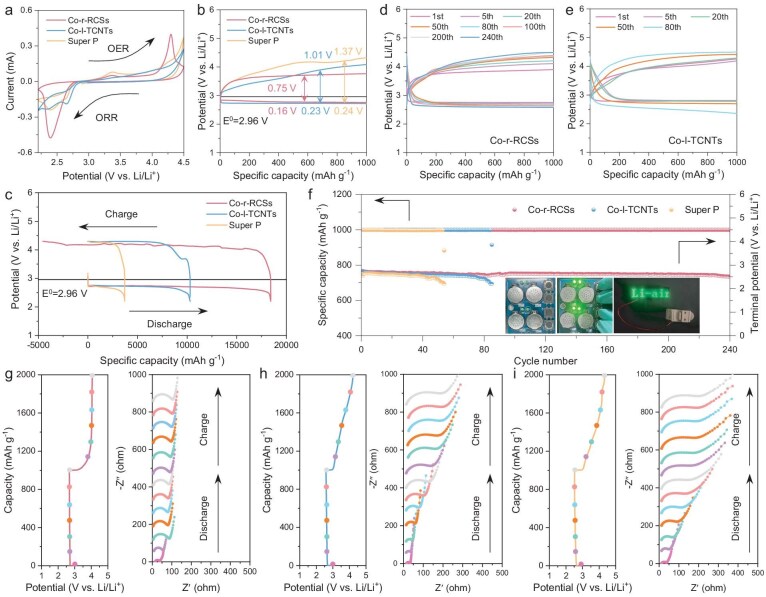
(a) CV curves of various electrodes at a scan rate of 0.2 mV s^−1^. The discharge-charge curves of various electrodes at (b) fixed capacity of 1000 mAh g^−1^ (2.2–4.5 V) and (c) full capacity (2.2–4.3 V) at a current density of 100 mA g^−1^. (d–f) Cycling stability and terminal discharge-charge voltages of various electrodes at 400 mA g^−1^ with a fixed capacity of 1000 mAh g^−1^. Inset: photo image of the LED turned on by four and two fabricated Co-r-RCSs-based Li-O_2_ button batteries, respectively. *In-situ* EIS spectra during discharge/charging in LOBs with (g) Co-r-RCSs, (h) Co-l-TCNTs and (i) Super P electrodes. The electrolyte used was 1 M lithium bis(trifluoromethane)sulfonamide in tetraethylene glycol dimethyl ether (LiTFSI/TEGDME).

To demonstrate the distinct electrocatalytic abilities of the two catalysts for the ORR/OER processes, as well as to elucidate their specific roles in the nucleation and decomposition of Li_2_O_2_, we conducted a comprehensive set of electrochemical tests. To ensure a systematic comparison, three oxygen electrodes were employed: Co-r-RCSs, Co-l-TCNTs and Super P. Figure 3a exhibits the cyclic voltammogram (CV) profiles of these three samples, recorded at a scan rate of 0.2 mV s^−1^ within a voltage window of 2.2–4.5 V. During the cathodic sweep, all electrodes exhibit a reduction peak corresponding to the ORR process, which is associated with Li_2_O_2_ nucleation. Conversely, during the anodic scan, distinct oxidation peaks emerge, signifying the bulk decomposition process of the discharged products. The Co-r-RCSs electrode shows significantly higher cathodic/anodic current peaks, along with a larger integrated area, indicative of higher specific capacitance compared to the other two electrodes. This suggests that the Co-r-RCSs electrode facilitates a more efficient nucleation and reversible decomposition of discharged products, thereby indicating significantly enhanced catalytic kinetics. The overpotential during the OER process for Co-l-TCNTs and Super P was evaluated using the first discharge/charge process at a fixed capacity of 1000 mAh g^−1^ and a current density of 100 mA g^−1^. As shown in Fig. 3b, the voltage gap for Co-l-TCNTs and Super P was determined to be 1.24 V and 1.61 V, respectively, whereas the Co-r-RCSs exhibited a significantly lower voltage gap of 0.91 V. This substantial difference in performance between Co-r-RCSs and Co-l-TCNTs can be primarily attributed to their surface structures. For the Co-r-RCSs electrode, the presence of a thin carbon coating on the surface exposes more metal active sites, thereby enhancing catalytic activity during the ORR/OER process [29]. This effect is further substantiated by ECSA calculations (details provided in the supplementary data) [24,30]. The calculated ECSA value of Co-r-RCSs (9.65 cm^2^_ECSA_) surpasses that of both Co-l-TCNTs (8.58 cm^2^_ECSA_) and Super P (4.64 cm^2^_ECSA_), further confirming the superior electrocatalytic activity of Co-r-RCSs. The galvanostatic charge/discharge (GCD) profiles of the three electrodes during deep discharge/charge cycles are presented Fig. 3c. Among them, the Co-r-RCSs delivers the highest capacity of 18 429.6 mAh g^−1^, which is more than five times greater than that of Super P (3715.6 mAh g^−1^), and also significantly higher than that of Co-l-TCNTs (10 308.2 mAh g^−1^). The Co-r-RCSs electrode catalyzes the bulk Li_2_O_2_→Li_2−x_O_2_→LiO_2_→O_2_ multi-step decomposition reaction during the charging process, showing a lower charge overpotential than the other two electrodes, while providing more capacity compared to its full discharge capacity before reaching the cut-off potential (4.3 V), mainly due to the decomposition of electrolyte.

Furthermore, under a fixed specific capacity of 1000 mAh g^−1^ at a current density of 400 mA g^−1^, the LOB with Co-r-RCSs exhibits remarkable cycling stability, sustaining over 240 cycles (Fig. 3d and f). In contrast, the Co-l-TCNTs and Super P fail to recover their discharged capacity when charged to 4.5 V, leading to rapid degradation, and only operate for 80 and 40 cycles, respectively (Fig. 3e and Fig. S26). The long cycling stability of Co-r-RCSs can be attributed to efficient spin-related charge transfer and the inhibition of side reactions, [31,32] which will be further discussed in subsequent sections. To further highlight its practical potential, the insets of Fig. 3f and Fig. S27 illustrate the successful operation installation of Li-O_2_ button batteries using a Co-r-RCSs electrode to power both a single light-emitting diode (LED) and an LED array in an ambient air atmosphere. The initial open circuit voltage of a single battery is greater than 3 V. The rate capability of the Co-r-RCSs-based LOB is displayed in Fig. S28, demonstrating a strong ability to recover its performance when the current density is increased from 100 to 1000 mA g^−1^ and then returned to 100 mA g^−1^. Conversely, Co-l-TCNTs and Super P fail to withstand the maximum current density of 1000 mA g^−1^ (Figs S29 and S30). Thus, a comprehensive suite of electrochemical tests prove that Co-r-RCSs display superior rate capability and cycling performance. Figure 3g–i present the *in-situ* electrochemical impedance spectroscopy (EIS) measurements of LOBs with the three electrodes during discharge and charge. The formation of an insulating Li_2_O_2_ layer is the primary cause of impedance increase [11]. Among the three electrodes, the Co-r-RCSs electrode shows the smallest impedance rise, indicating that most of the Li_2_O_2_ forms in solution rather than passivating the oxygen electrode.

### Microstructure evolution of products during ORR/OER processes

**Figure 4.**
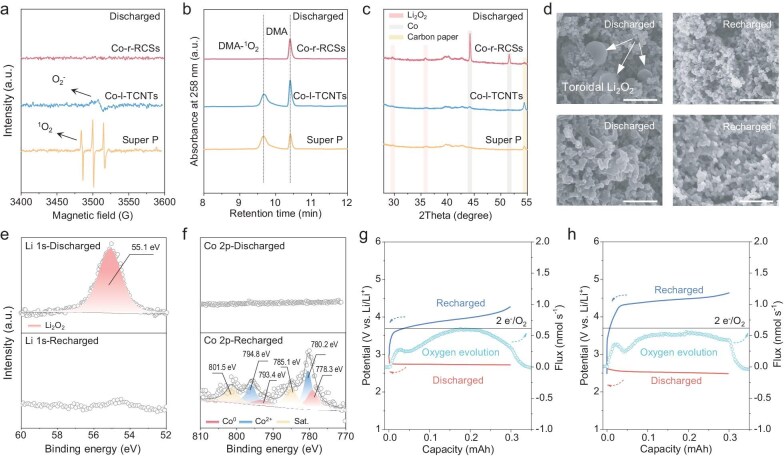
(a) *Ex-situ* EPR spectra of the separators and (b) HPLC analyses of the electrolyte extracted from the discharged coin batteries with various electrodes. (c) *Ex-situ* XRD patterns of various electrodes after discharging. (d) SEM of Co-r-RCSs (top) and Co-l-TCNTs (bottom) after discharging and recharging (scale bar, 1 μm). (e) Li 1s and (f) Co 2p XPS fitting of the Co-r-RCSs after discharging and recharging. The *in-situ* DEMS analysis of the gas evolution during the charge process of LOBs operation with (g) Co-r-RCSs and (h) Co-l-TCNTs. All electrodes were tested after the first cycle at a fixed capacity of 1000 mAh g^−1^ and a current density of 400 mA g^−1^.

A thorough comprehension of the ORR/OER mechanism is crucial for guiding future catalyst design. One of the key aspects is to elucidate the evolution of discharge and charge products on the oxygen electrodes. The O_2_-related intermediates (O_2_^−^, ^1^O_2_) in LOBs with different electrodes were monitored using *ex-situ* electron paramagnetic resonance (EPR) spectroscopy and high-performance liquid chromatography (HPLC) [32,33]. In Fig. 4a, no detectable signal was observed for discharged Co-r-RCSs, which is consistent with that of the pristine separator (Fig. S31). In contrast, clear signals corresponding to O_2_^−^ and ^1^O_2_ were detected in discharged Co-l-TCNTs and Super P electrodes, [32,34] with Super P exhibiting a particularly high concentration of ^1^O_2_. Additionally, EPR analysis of the recharged Super-P-based-battery revealed a strong Li metal signal, [35] while severe corrosion was observed on the Li electrode surface (Fig. S32 and S33). This degradation can be attributed to the highly active ^1^O_2_ species, which promotes parasitic reactions, leading to the formation of Li dendrites and their subsequent accumulation on the separator. Figure 4b shows HPLC-based detection of the conversion of 9,10-dimethylanthracene (DMA) to its coordination product (DMA-^1^O_2_) in LOBs with different electrodes after discharge. A high concentration of 30 mM DMA was added to the electrolyte to ensure complete ^1^O_2_ capture. Significant signals of DMA (retention time: 10.4 min) and DMA-^1^O_2_ (∼9.7 min) were observed for Co-l-TCNTs and Super P electrodes. The ^1^O_2_ primarily originates from the disproportionation of LiO_2_. The detailed reaction is as follows [36,37]:


(5) \begin{eqnarray*}
{\mathrm{2Li}}{{\mathrm{O}}_{\mathrm{2}}} \to {\mathrm{L}}{{\mathrm{i}}_{\mathrm{2}}}{{\mathrm{O}}_{\mathrm{2}}} + {{\mathrm{x}}^{\mathrm{3}}}{{\mathrm{O}}_{\mathrm{2}}} + {\mathrm{ (1 - x}}{{\mathrm{)}}^{\mathrm{1}}}{{\mathrm{O}}_{\mathrm{2}}}.
\end{eqnarray*}

Interestingly, after the recharge process, the DMA-^1^O_2_ signals of Co-l-TCNTs and Super P nearly disappeared (Fig. S34), indicating that ^1^O_2_ was consumed in side reactions during charging. However, no detectable ^1^O_2_ signals were observed in Co-r-RCSs-based LOBs throughout the discharge and charge process. To further validate these findings, the evolution of O_2_ release during charging was monitored (Fig. S35). The Co-r-RCSs electrode-based LOB exhibited higher O_2_ evolution, indicating that the occurrence of parasitic side reactions was effectively suppressed.

As depicted in Fig. 4c, *ex-situ* XRD analysis of the three electrodes reveals that Co-r-RCSs exhibits new characteristic peaks corresponding to the Li_2_O_2_ (JCPDS Card No. 44-1485), which completely disappear upon recharging (Fig. S36) [38]. This result indicates that the nucleation and decomposition of Li_2_O_2_ contribute predominantly to the overall capacity. However, for the discharged Co-l-TCNTs and Super P electrodes, the characteristic peaks of Li_2_O_2_ are significantly less pronounced, suggesting that their sluggish ORR/OER kinetics hinder the efficient nucleation and decomposition of Li_2_O_2_. To further investigate the morphological differences, *ex-situ* SEM analysis was performed to track the evolution of the electrode surfaces. As shown in Fig. 4d, large toroidal Li_2_O_2_ particles are uniformly distributed on the surface of Co-r-RCSs after discharging. Upon recharging, these particles fully disappear, restoring a uniformly dispersed catalyst distribution. In contrast, the surface of the Co-l-TCNTs electrode is covered with a thin film-like reduction product after discharging, which remains partially intact even after recharging. Combining the morphological features, *ex-situ* EPR and HPLC results of the discharge products, it can be inferred that Co-r-RCSs facilitates a solution-phase growth pathway for toroidal Li_2_O_2_, whereas the Co-l-TCNTs promotes a mixed mechanism involving both surface-mediated and partial solution-phase growth [4]. Moreover, almost no obvious products were observed on the surface of Super P, yet severe electrode surface passivation occurs after recharging (Fig. S37). Figure 4e and f, and Figs S38–S40 present comprehensive *ex-situ* XPS and FTIR evidence of the cycled electrodes. For Co-r-RCSs, the distinct peaks observed in the Li 1s and Co 2p spectra provide clear evidence of the reversible nucleation and decomposition of Li_2_O_2_ during the discharge/recharge process. Furthermore, characteristic peaks associated with by-products are scarcely detected, indicating minimal parasitic reactions. The increase in the Co^2+^ content in recharged Co-r-RCSs electrode is attributed to the strong adsorption of O_2_ on the surface of the active sites in the saturated O_2_ environment, which leads to the inevitable oxidation of Co. In contrast, after discharging, the Li 1s spectra of Co-l-TCNTs and Super P exhibit prominent signals corresponding to the by-product Li_2_CO_3_ at ∼56.0 eV [39]. Additionally, both Li_2_CO_3_ and Li_2_O_2_ are incompletely decomposed even after recharging, suggesting substantial side reactions and sluggish OER kinetics. Moreover, for Co-l-TCNTs, the Co 2p profile shows only weak Co-related signals after recharging, indicating that the electrode surface remains largely covered by residual discharged products or by-products. This observation is consistent with *ex-situ* XRD and SEM analyses, reinforcing that Co-l-TCNTs and Super P exhibit poor reversibility. Meanwhile, FTIR analysis confirms that Co-r-RCSs accumulates fewer side products over cycles than the other electrodes (Fig. S40). The OER process was further studied using quantitative *in-situ* differential electrochemical mass spectrometry (DEMS). During the charging process, O_2_ evolution initially increases, followed by a sharp decline, and then rises again in the mid-charging stage (Fig. 4g and h, and Fig. S41). This trend corresponds to the typical decomposition of Li_2_O_2_ accompanied by side reactions that lead to O_2_ loss [40,41]. Compared to Co-l-TCNTs, the O_2_ evolution in the Co-r-RCSs-based LOB shows a steady profile from the onset of charging, with a significantly reduced ORR/OER overpotential. Fig. S42 shows the integrated O_2_ release-to-charge and charge-to-O_2_ ratios during the charge process, suggesting an approximate 2e^−^ reaction for Li_2_O_2_ decomposition in the Co-r-RCSs electrode. The higher charge-to-O_2_ ratios observed for the Co-l-CNTs and Super P electrodes indicate the occurrence of side reactions, such as oxidation of C with Li_2_O_2_, or that the decomposition of Li_2_CO_3_ occurs during the charging process [42].

### Spin-related electron transfer and density functional theory calculations

**Figure 5.**
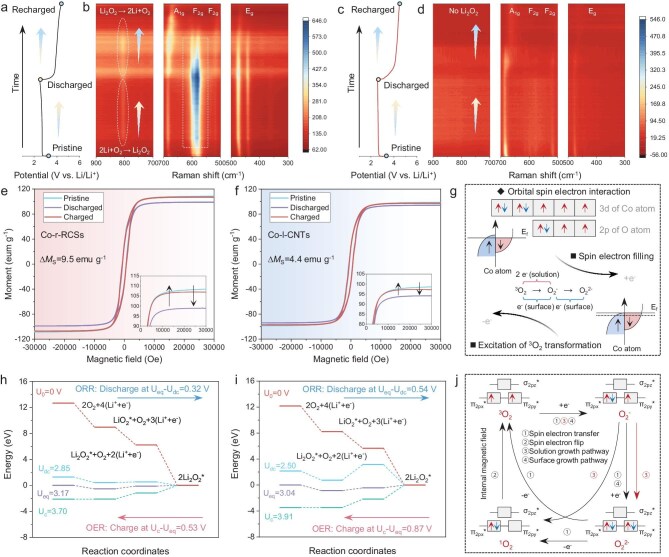
Discharge/charge curves and corresponding *in-situ* Raman contour diagram or spectra of (a and b) Co-r-RCSs and (c and d) Co-l-TCNTs electrodes. (e and f) Magnetic hysteresis measured at corresponding states (first cycle at a fixed capacity of 1000 mAh g^−1^ and a current density of 400 mA g^−1^) with an applied magnetic field of 3 Tesla. (g) The schematic diagram of the reaction process and the corresponding electron changes at the Fermi level of the Co atom. The Gibbs free energy at zero, equilibrium, discharge and charge voltages for (h) Co-r-RCSs and (i) Co-l-TCNTs. Asterisks denote the species adsorbed on the surface of catalysts. (j) The electronic configuration changes of O_2_-related species during the corresponding reactions.

To investigate the dynamic evolution of electron transfer states during the ORR/OER process, *in-situ* Raman spectroscopy analysis was conducted. A comparative analysis reveals that Li_2_O_2_ (808 cm^−1^) on the Co-r-RCSs electrode undergoes reversible nucleation and decomposition throughout the discharge/charge cycle (Fig. 5a and b, and Fig. S43). However, no Li_2_O_2_ nucleation was observed on the Co-l-TCNTs electrode (Fig. 5c and d, and Fig. S44). Moreover, the electronic structure of the catalyst exhibits distinct transformations. For Co-r-RCSs, the intensity of the E_g_, F_2 g_ and A_1 g_ vibration peaks of Co-O shows consistent enhancement and weakening trend during Li_2_O_2_ nucleation and decomposition. The E_g_ and F_2 g_ orbitals of Co metal/oxide initially exhibit splitting, indicating strong O_2_ adsorption in an oxidizing environment, leading to surface restructuring [43]. This charge redistribution is believed to optimize the adsorption of O-containing intermediates, thereby enhancing catalytic activity [44]. In addition, the A_1 g_ orbital of Co-O undergoes an initial blue shift followed by a pronounced red shift, indicating good reversibility [43]. These spectral changes indicate that the Co-r-RCSs electrode can sustain high catalytic activity under demanding ORR/OER conditions. In contrast, the signal intensities of the E_g_, F_2 g_ and A_1 g_ vibration peaks in Co-l-TCNTs continuously weaken, eventually blurring in the contour map. This suggests that the Co-l-TCNTs catalyst dissolves in a strong oxidizing environment, leading to severe electrode degradation [21].

To elucidate the spin-related electron transfer mechanism during the ORR/OER process, *ex-situ* magnetometry was performed, as shown in Fig. 5e and f. Clearly, the M_S_ of both electrodes decreases after discharging and returns to nearly its initial value after recharging. The decrease in M_S_ for Co-r-RCSs was 9.5 emu g^−1^, more than twice that of Co-l-TCNTs (∆M_S _= 4.4 emu g^−1^), indicating a significantly higher degree of spin polarization. The net magnetization of TM is defined as M = (N_↑_−N_↓_) × *μ*_B_, where N_↑_ and N_↓_ represent the total number of spin-up and spin-down d-band electrons, and *μ*_B_ is the Bohr magneton [45]. During discharging, spin-polarized electrons accumulate near the Fermi level of the FM Co, facilitating rapid coherent spin exchange between the orbitals of Co atom and O_2_ molecules [46]. The significant decrease in M_S_ of the Co-l-TCNTs electrode after discharge suggests a redistribution of d-orbital electrons, leading to a reduced disparity between the number of N_↑_ and N_↓_ electrons. The designed Co-r-RCSs has a lower d-band center, allowing it to accommodate more spin-filled electrons. This provides sufficient electron transfer for the first RDS, thereby enabling an efficient solution-phase reduction of ^3^O_2_ (^3^O_2_ → O_2_^−^ → O_2_^2−^), as illustrated in Fig. 5g. During the OER process, electron transfer follows the Hund's rule and Pauling exclusion principle, leading to spontaneous spin polarization [46]. Most of the prefilled spin electrons in Co are extracted and re-excited to generate ^3^O_2_, enabling the M_S_ of the catalyst to return to its initial state.

Figure 5h and i illustrate the calculated Gibbs free energy diagrams for the evolution of the discharged products during the ORR/OER on the (111) surfaces of Co-r-RCSs and Co-l-TCNTs, respectively. The overpotentials for ORR and OER processes are defined as η_ORR _= U_dc_−U_eq_ and η_OER _= U_c_−U_eq_, where U_eq_ represents the equilibrium potential, while U_dc_ and U_c_ correspond to the discharge and charge potentials [47], respectively. For Co-r-RCSs, the calculated equilibrium potential is 3.17 V, corresponding to the ORR and OER overpotentials of 0.32 and 0.53 V. In contrast, Co-l-TCNTs exhibits a lower equilibrium potential of 3.04 V, with significantly higher overpotentials of 0.54 (ORR) and 0.87 V (OER). This suggests that Co-r-RCSs can efficiently promote the nucleation/decomposition of Li_2_O_2_ during ORR/OER processes [48,49]. The specific electron transfer between O_2_-containing species during the ORR/OER process are illustrated in Fig. 5j. In the first RDS, electrons are transferred from the electrode to the ^3^O_2_ π* orbital, forming O_2_^−^. The electrical conductivity of catalysts and their adsorption behavior toward ROS are critical factors in determining the reaction pathway of the second step, whether it follows a solution growth pathway or surface growth pathway [28]. The solution-phase reaction pathway will lead to the formation of highly reactive ^1^O_2_, which aggressively attacks both the electrolyte and Li electrode, resulting in poor battery cycle stability [33]. Converting ^1^O_2_ back to ^3^O_2_ requires additional energy for spin flip. It has been reported that electron-rich RMs in the electrolyte can facilitate this conversion via spin flipping to quench ^1^O_2_ [50]. Here, the internal magnetic field of FM-ordered Co-r-RCSs quickly drives the spin-flip effect, achieving the conversion of ^1^O_2_ to ^3^O_2_ at the electrode/electrolyte interface, [17] and ^3^O_2_ cannot be converted to high-energy ^1^O_2_ by the spin-flip effect.

## CONCLUSION

In summary, this study systematically investigated the role of selective d-orbital filling in oxygen electrodes and its influence on the nucleation and decomposition of Li_2_O_2_ in LOBs. The Co-r-RCSs catalyst, with its rationally designed composition and electronic structure, exhibits enhanced catalytic activity even under strongly oxidative conditions. During discharge, a significant accumulation of spin-polarized electrons near the Fermi level of Co atoms facilitates efficient spin-related electron exchange between Co orbitals and O_2_ molecules. Magnetic measurements further revealed a pronounced decrease and subsequent recovery of M_S_ in Co-r-RCSs during the discharge/charge process, indicating dynamic spin-state transitions. The lower d-band center of Co 3d orbitals in Co-r-RCSs weakens the binding interaction with LiO_2_, thereby promoting selective electron transfer to stabilize LiO_2_ in solution. This mechanism guides the solution-phase growth of Li_2_O_2_, significantly enhancing the reversibility of Li_2_O_2_ nucleation and decomposition. Moreover, the internal magnetic field of Co-r-RCSs can inhibit the ^1^O_2_ formation through spin flipping, thereby mitigating parasitic reactions. As a result, Co-r-RCSs exhibits superior rate performance, extended cycling stability and reduced overpotential in LOBs. This study elucidates the spin-related electron catalysis mechanism of Li_2_O_2_ nucleation/decomposition in LOBs and contributes to the rational design of high-performance catalysts.

## Supplementary Material

nwaf145_Supplemental_File
