# Supplementary material for: Spin-polarized d-orbital filling in cobalt catalysts boosts solution-mediated Li-O2 batteries
Source: Natl Sci Rev. 2025 Apr 24;12(6):nwaf145. doi: 10.1093/nsr/nwaf145 (PMC12064849; doi:10.1093/nsr/nwaf145)
Supplement: nwaf145_Supplemental_File [file nwaf145_supplemental_file.pdf]

Supplementary Information

Spin polarized d-orbital filling in cobalt catalysts boosts solution-mediated Li-O_2 batteries

Fengling Zhang,[1] Zhengqiang Hu,[1] Jingning Lai,[1,*] Nuo Chen,[1] Yuhao Liu,[1] Tianyang Yu,[1] Faiza Arshad,[1] Liyuan Zhao,[1] Nan Chen,[1,2,3] Li Li,[1,2,3,4] Qiang Li,[5] Feng Wu[1,2,3,4] and Renjie Chen[1,2,3,4,*]

[1]Beijing Key Laboratory of Environmental Science and Engineering, School of Materials Science & Engineering, Beijing Institute of Technology, Beijing 100081, China;

[2]Innovative Research Team in High-Safety Energy Storage System and Smart Microgrids of Guangdong Province, Beijing Institute of Technology (Zhuhai), Zhuhai 519088, China;

[3]Shandong Key Laboratory of Advanced Chemical Energy Storage and Intelligent Safety, Advanced Technology Research Institute, Beijing Institute of Technology, Jinan 250300, China;

[4]Collaborative Innovation Center of Electric Vehicles in Beijing, Beijing 100081, China;

[5]College of Physics, Weihai Innovation Research Institute, Institute of Materials for Energy and Environment, Qingdao University, Qingdao 266071, China

[*]**Corresponding authors.** E-mails: chenrj@bit.edu.cn; laijn@bit.edu.cn

1. Experimental Materials

Polyvinylidene fluoride (PVDF, 99.999%), N-Methyl pyrrolidone (NMP, 99.9%), Cobalt (II) nitrate hexahydrate ($Co(NO_3)_2 \cdot 6H_2O$, 99%), potassium hexacyanocobaltate(III) ($K_3[Co(CN)_6]$, 98%) were purchased from Aladdin (China). Super P (Canrd Technology Co. Ltd.) was used as air electrode and conductive agent. Li foil (Canrd Technology Co. Ltd.) was used as negative electrode. 1 M lithium bis(trifluoromethane)sulfonamide/tetraethylene glycol dimethyl ether (LiTFSI/TEGDME) was purchased from Suzhou Duoduo Chemical Technology Co. TO (>99% purity). 9,10-dimethylanthracene (DMA, Adamas-beta®), dimethoxyethane (DME, Aladdin) and 2,2,6,6-tetramethyl-4-piperidone (4-Oxo-TEMP, Aladdin) were directly used without further treatment. All chemicals were used as received without further purification.

2. Synthesis of Co-r-RCSs and Co-l-TCNTs

To synthesize the Co-l-TCNTs catalyst, 2.1859 g $Co(NO_3)_2 \cdot 6H_2O$ was dissolved in 100 mL deionized water to form solution 1, 1.6676 g $K_3[Co(CN)_6]$ was dissolved in 100 mL deionized water to form solution 2, then solution 1 was slowly dripped into solution 2 to form a cloudy pink solution, which was continuously stirred at room temperature at 400 rpm for 24 h. The uniformly stirred solution was centrifuged with deionized water for 3 times, and then dried in a freeze dryer for 24 h. Subsequently, the dried purple sample was annealed, and the temperature was raised to 700 ℃ at a heating rate of 5 ℃/min in an Ar atmosphere. After holding for 3 h, the Co-l-TCNTs sample was obtained after it was cooled to room temperature. To obtain a catalyst with reduced and suitable surface carbon coating thickness, the other conditions are consistent with the synthesis steps of Co-l-TCNTs, while the annealing treatment is carried out differently. First, it was heated to 550 ℃ at a rate of 5 ℃/min for 1 h to stabilize carbonization process, then continued at this heating rate to 900 ℃ (Co-r-RCSs catalyst) or 1100 ℃ for 2 h, and finally cooled down to room temperature.

3. Materials Characterization

Powder X-ray diffraction (XRD) analysis was carried out with a diffractometer with Cu Kα radiation (Rigaku D/Max-KA, λ=1.5406 Å). The morphology features were observed based on scanning electron microscope (SEM) (Hitachi S-4800), transmission electron microscopy (TEM) and X-ray energy dispersive spectroscopic (EDS)-mapping (JEOL JEM-ARM200CF). X-ray photoelectron spectroscopy (XPS) experiments were carried out on PHI-1600 ESCA system using Al Kα radiation. The specific surface area of the samples was confirmed using the Brunauer-Emmett-Teller (BET) method by N_2 adsorption and desorption tests using the Quadrasorb SI analyzer at 77 K. The molecular orbital characteristics of samples were collected via a micro-Raman spectrometer (Thermo Fisher DXR2) by a 532 nm laser. In-situ Raman spectroscopy tests were carried out with an in-situ Raman

battery mold (Beijing Science Star Technology Co., Ltd China) to assemble the Li-O_2 battery. Fourier transform infrared spectrometer (FTIR) spectroscopy was conducted on a Autosorb-iQ system. The pre-washed electrodes were transferred into the chamber in an Ar-filled glovebox to avoid air exposure. The X-ray absorption near-edge structure (XANES) of Co K-edge were monitored at beamline BL02B of Shanghai Synchrotron Radiation Facility using the transmission mode. The magnetic properties were probed by a Quantum Design physical property measurement system (PPMS) magnetometer and vibrating sample magnetometry (VSM) at room temperature (300 K) with an applied magnetic field of 3 tesla.

4. Electrochemical Measurements

The working electrode was prepared by casting the well agitated slurry and drying at 100 ℃ for 24 h, containing 40% catalysts and 40% Super P and 20% PVDF dissolved in NMP onto carbon paper. The mass loading of average oxygen electrode is 0.2-0.4 mg cm^{-2}. Then, the Li-O_2 batteries (CR2032 coin cells) were assembled in an Ar-filled glovebox (the content of H_2O and O_2 below 0.01 ppm). The total mass of battery is about 3.6 g. Concretely, Whatman glass fiber separator (GF/D) was placed between catalytic electrode and Li electrode with 150 μL electrolyte, the electrolyte was 1 M lithium bis(trifluoromethane)sulfonamide/tetraethylene glycol dimethyl ether (LiTFSI/TEGDME). All the batteries were tested at room temperature with a potential range of 2.2-4.5 V (vs Li/Li^+). GCD data were recorded in an O_2-purged tube using multichannel battery systems instrument (LAND CT 2001A and Neware CT-4008T). CV measurements at a scan rate of 0.2 mV s^{-1} and Electrochemical impedance spectroscopy (EIS) tests were performed on an electrochemical workstation (Corrtest Instruments and CHI 760E).

5. In-situ differential electrochemical mass spectrometry (DEMS) measurement

In-situ DEMS measurement was performed by electrochemical reactions, which conducted on a commercial quadrupole mass spectrometer (Hiden Analytical, model: HPR 40) and a Swagelok-type Li-O_2 cell. The assembled cell contained each oxygen electrode (Φ=11 mm) with loading density of 0.2-0.3 mg cm^{-2}, a piece of Li foil (Φ=16 mm), a glassy fiber separator (Φ=18 mm) with 100 μL of LiTFSI/TEGDME electrolyte. The cell was discharged with a mixed gas of Ar/O_2 (volume ratio: 4:1) as the working gas, and was charged with high-purity Ar as the working gas. All processes were linked to a mass spectrometer by a gas purging system. The flow rate of working/carrier gas (Ar/O_2 or Ar) was set at 0.5 mL min^{-1}. The evolution of O_2 was monitored by the mass spectrometer during charge.

6. Ex-situ $^1O_2$ Detection

Ex-situ electron paramagnetic resonance (EPR) experiments were performed to detect the released $^1O_2$, which were conducted on Burker EMX nano spectrometer with the frequency of 9.63 GHz and the

microwave power of 0.316 mW. 0.1 M 4-Oxo-TEMP was added in the 1 M LiTFSI/TEGDME electrolyte as a spin trap to capture $^1O_2$.[1] Each cell containing 100 uL electrolyte was discharged and charged to 1000 mAh g^{-1}, then disassembled to obtain the separator for ex situ EPR experiments, which was performed in an Ar-filled glove box and transfers the separator to a sealed quartz tube for testing. The $^1O_2$ detection was also determined through the transformation of DMA to DMA-O_2 using high performance liquid chromatography (HPLC) with a UV-vis detector (1200 series, Agilent Technologies).[2] 30 mM DMA was added in the 1 M LiTFSI/TEGDME electrolyte to capture the released $^1O_2$ and guarantee enhanced sensitivity. Each cell was discharge and charged to 1000 mAh g^{-1}, the electrolyte was extracted from the separator and electrodes using 400 μL DME inside an Ar-filled glovebox. Then the DME solution was evaporated in an Ar-filled glove box. After dissolving the residue in 100 μL DME, a volume of 2 μL was injected into the HPLC. The extent of the transformation of DMA to DMA-O_2 was measured at 258 nm.

7. Computational Details

Density functional theory (DFT) calculations were conducted using the Castep module in of the Materials Studio of Accelrys Inc.[3] The electron interactions were optimized by the Perdew-Burke-Ernzerhof (PBE) generalized gradient approximation.[4] To simulate the ratio of Co to O, we removed several oxygen atoms from both surface sides of the initial CoO (111) lattices. For the structural relaxations of the slabs, a vacuum slab of 15 Å is applied, in c-axis to eliminate the interplay between the periodic images. The convergence criterion for energy was limited at 1×10^{-5} eV. The planewave cut-off energy was controlled at 700 eV. The atomic geometries were relaxed until the threshold forces less than 0.05 eV $Å^{-1}$ during structure optimization. To address the strongly correlated and localized 3d shell of Co, we employed the DFT+Hubbard U (DFT+U) approach. U_{eff} = 4.1 eV on Co atoms was applied since the U_{eff} correction gives a lattice constant, magnetic moment, and band gap of CoO in good agreement with the experimental values.[5] The 3×3×1 k-point mesh set was used for Co-r-RCSs and Co-l-TCNTs models. The DFT-D2 method proposed by Grimme was chosen to describe the van der Waals interactions. The free energy was calculated based on the following equation:

$$G=E+ZPE-T_S$$

Where, G, E, ZPE and T_S are the free energy, total energy from DFT calculations, zero-point energy and entropic contributions (T was set to be 300 K), respectively.

8. Results and discussion

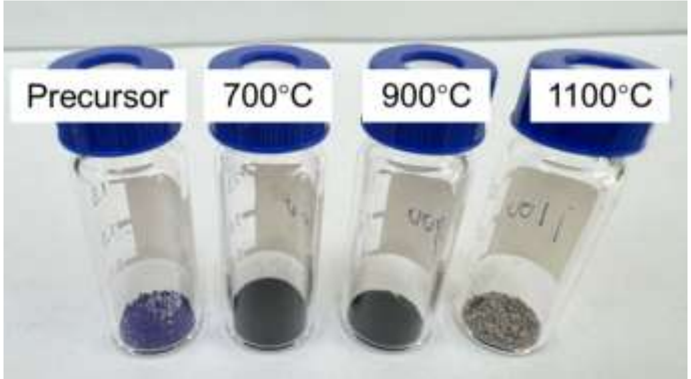

Figure S1. Photographs of the precursor samples and annealing at 700, 900, 1100℃, respectively.

It can be seen that the surface of the sample annealed at 1100℃ shows an obvious metallic luster without the presence of carbon, and the sample is a massive large particle that is hard and cannot be ground into slurry for coating. Therefore, this work mainly selects the samples annealed at 700℃ and 900℃ for research and comparison.

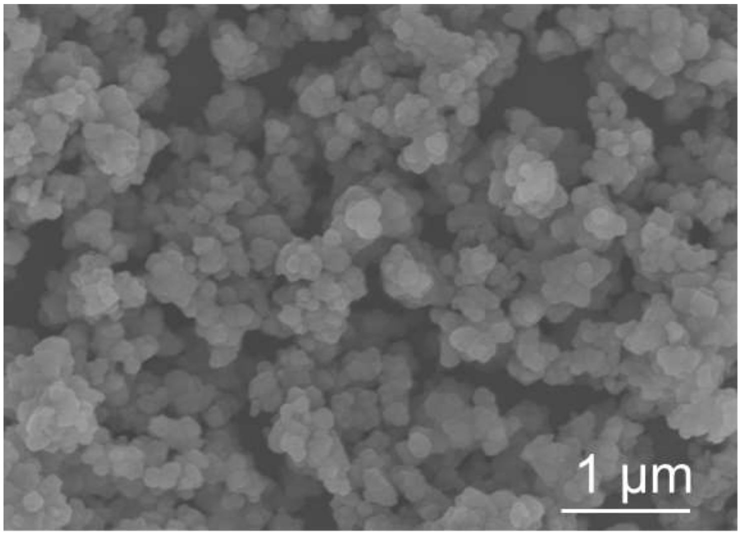

Figure S2. SEM of the precursor sample.

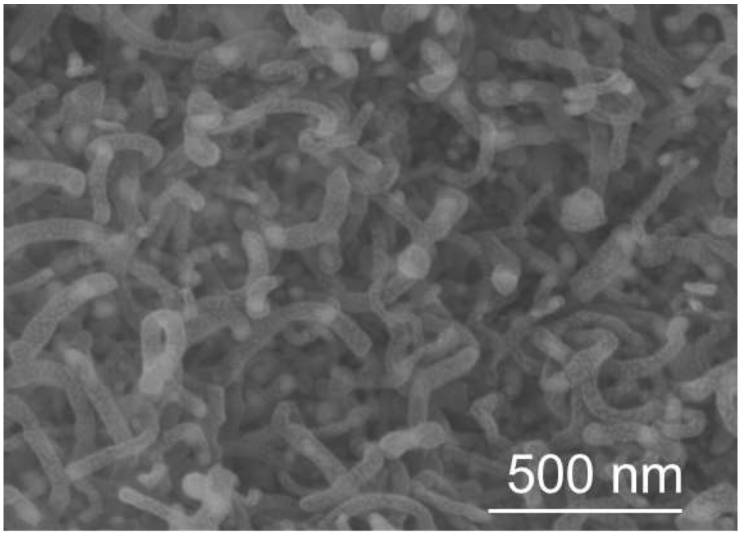

Figure S3. SEM of the Co-l-TCNTs sample.

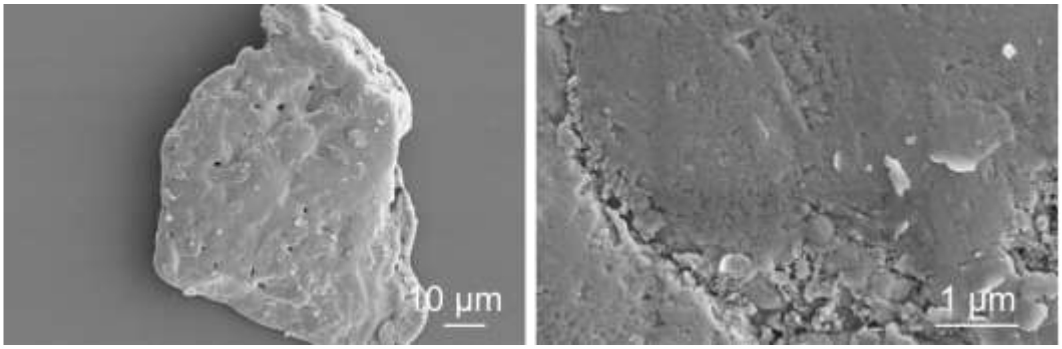

Figure S4. SEM of the sample annealed at 1100℃.

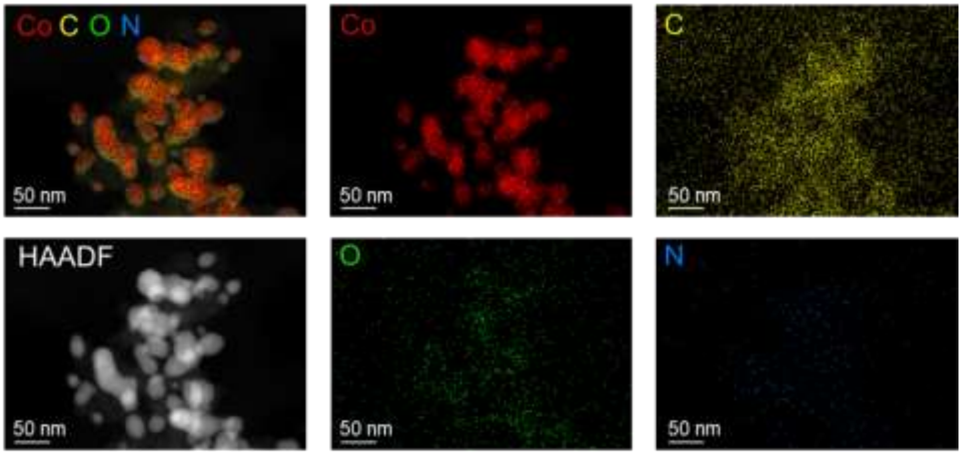

Figure S5. EDS-mapping images of Co-r-RCSs sample.

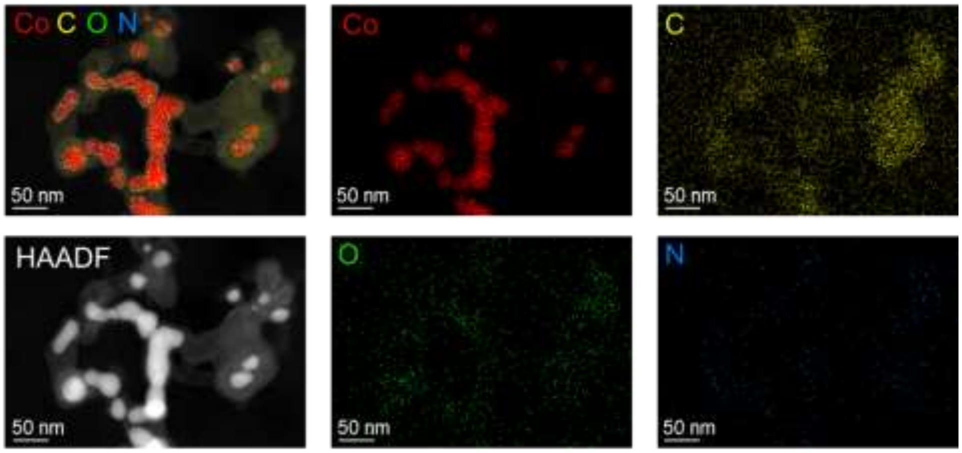

Figure S6. EDS-mapping images of Co-l-TCNTs sample.

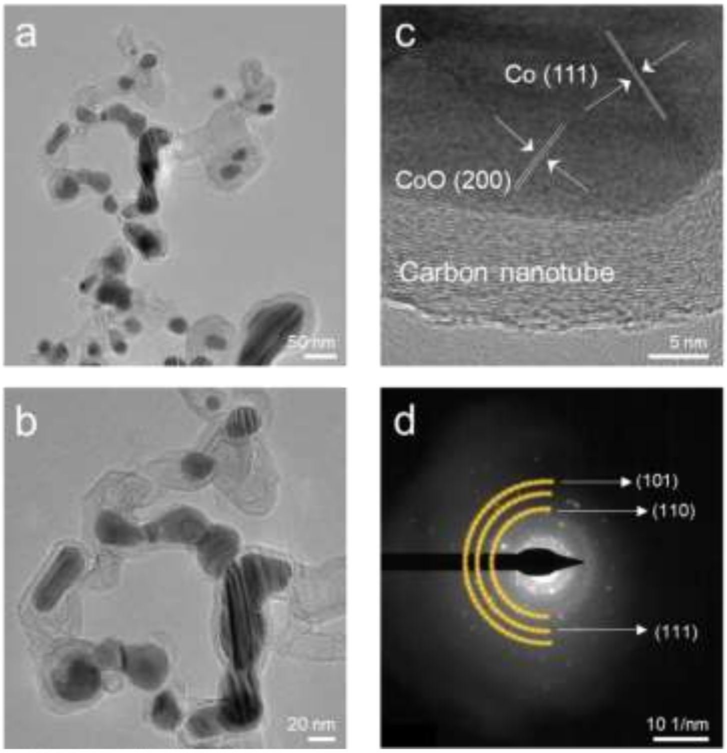

Figure S7. (a, b) TEM images, (c) HRTEM image and (d) SAED patterns of Co-l-TCNTs sample.

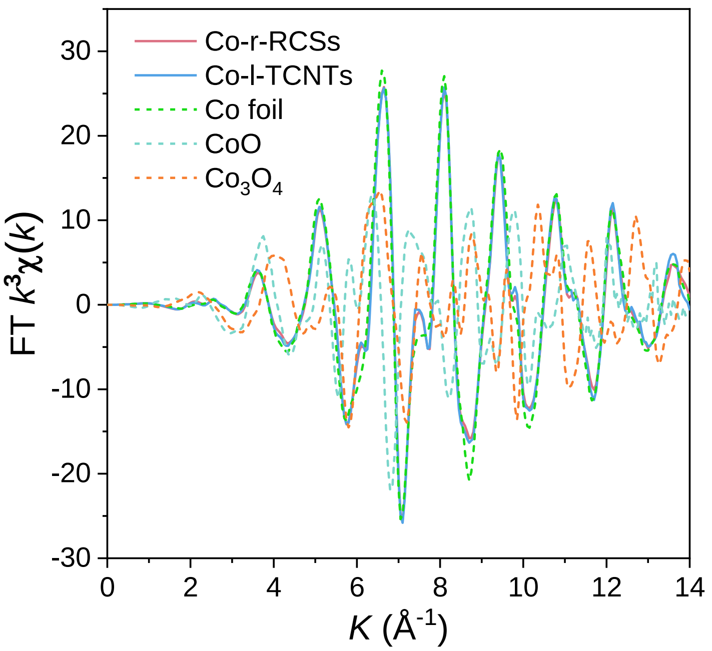

Figure S8. K-edge FT-EXAFS in k space for Co-r-RCSs, Co-l-TCNTs and Co-foil, CoO and Co_3O_4 reference samples.

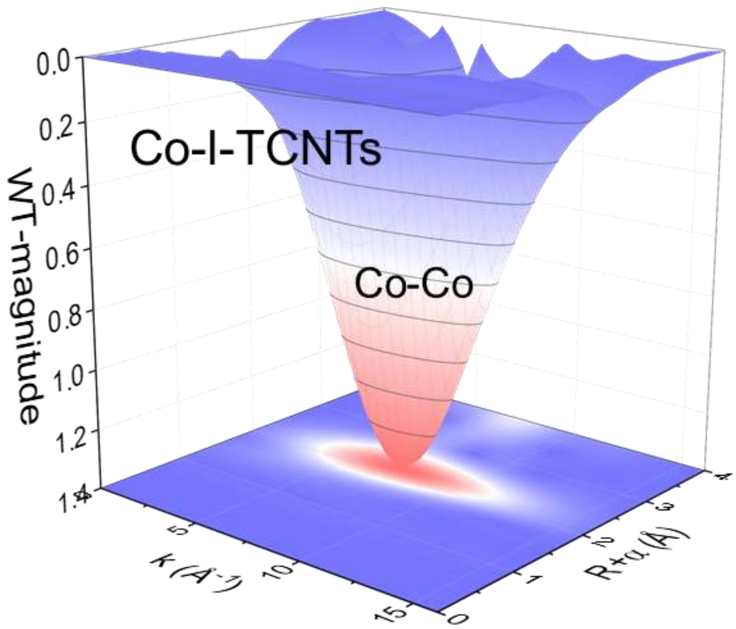

Figure S9. Wavelet transforms analysis of EXAFS signal of Co-l-TCNTs.

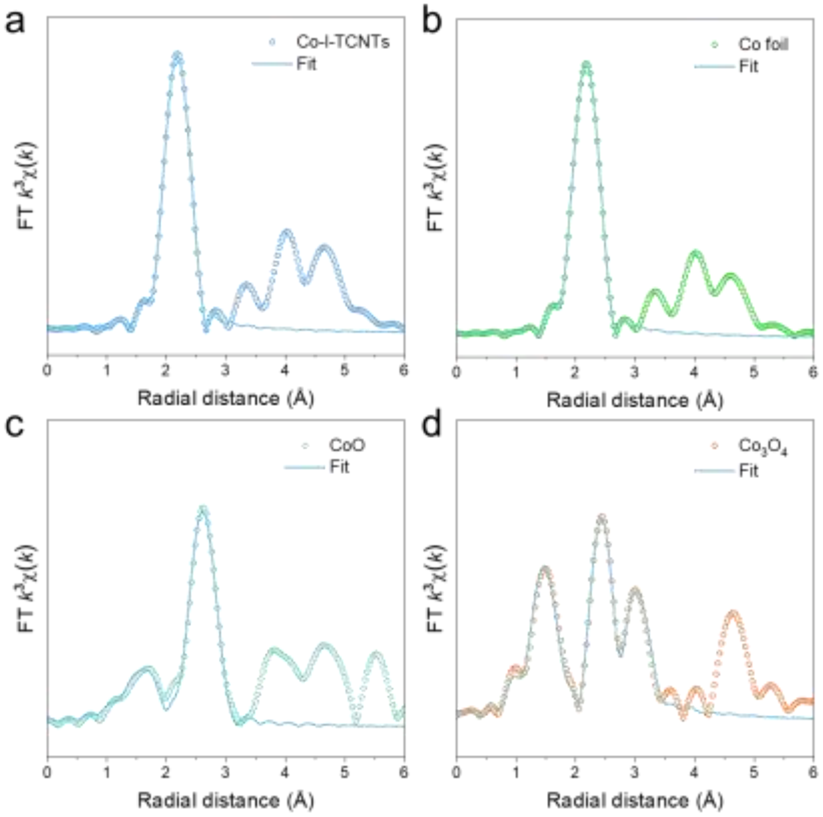

Figure S10. EXAFS fitting curves at R space for Co-l-TCNTs and Co-foil, CoO and Co_3O_4 reference samples.

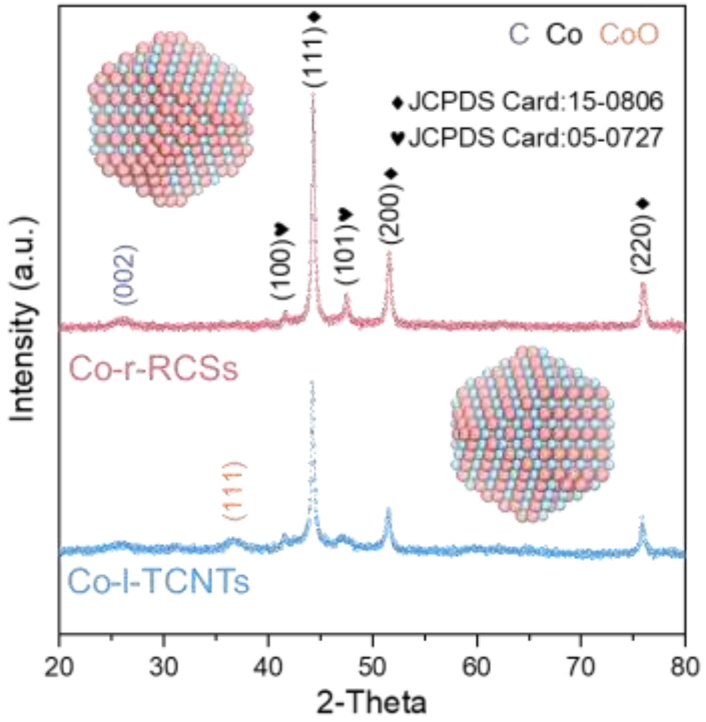

Figure S11. XRD spectra of Co-r-RCSs and Co-l-TCNTs.

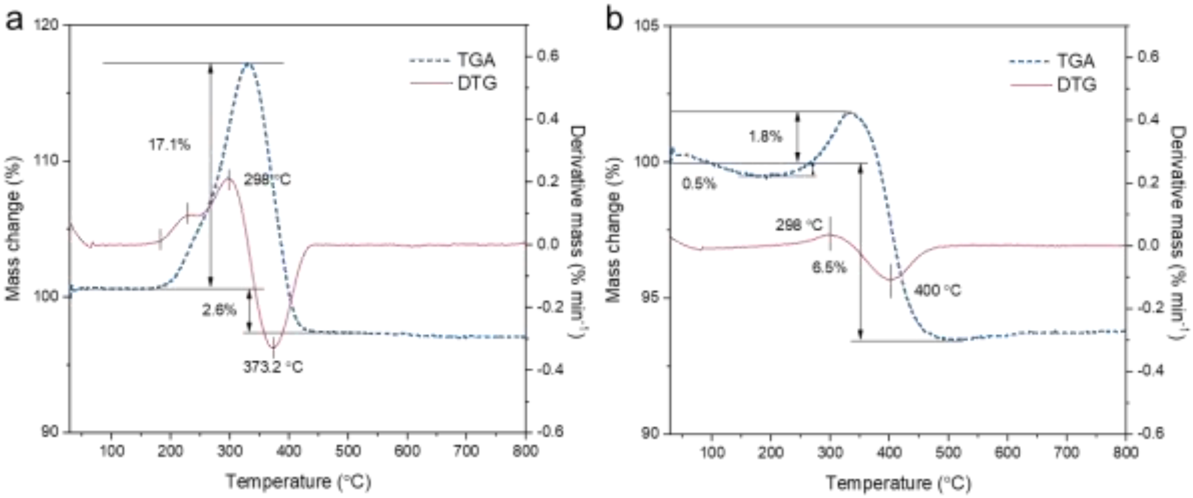

Figure S12. TG curves of (a) Co-r-RCSs and (b) Co-l-TCNTs samples carried out under air atmosphere with a heating rate of 5 ℃ min^{-1}.

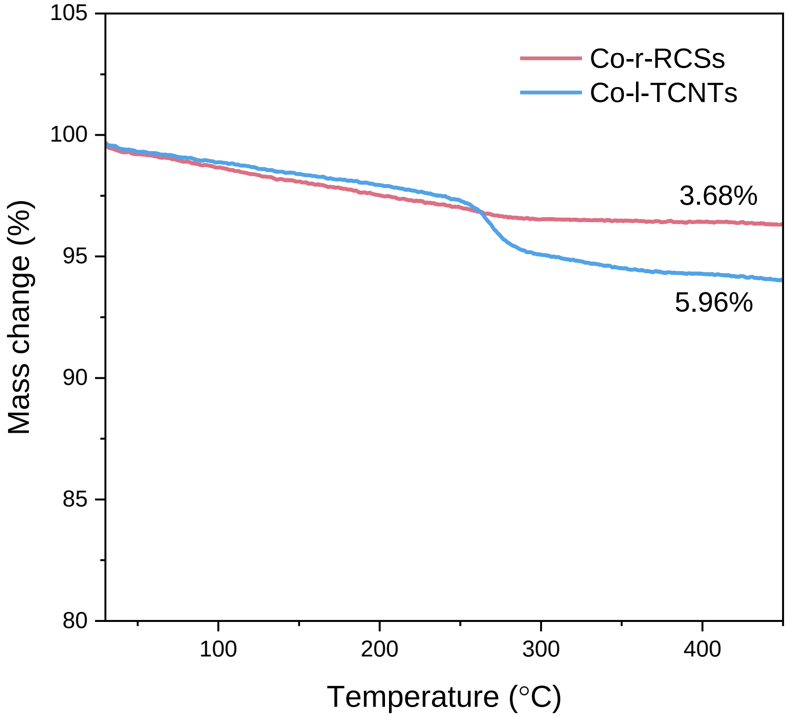

Figure S13. TG curves of Co-r-RCSs and Co-l-TCNTs samples carried out under Ar/H_2 (4 vol% H_2) with a heating rate of 5 ℃ min^{-1}. The calculated content of CoO is 17.25/27.94% from the loss of 3.68/5.96 wt% oxygen for Co-r-RCSs and Co-l-TCNTs samples in the TG profiles.

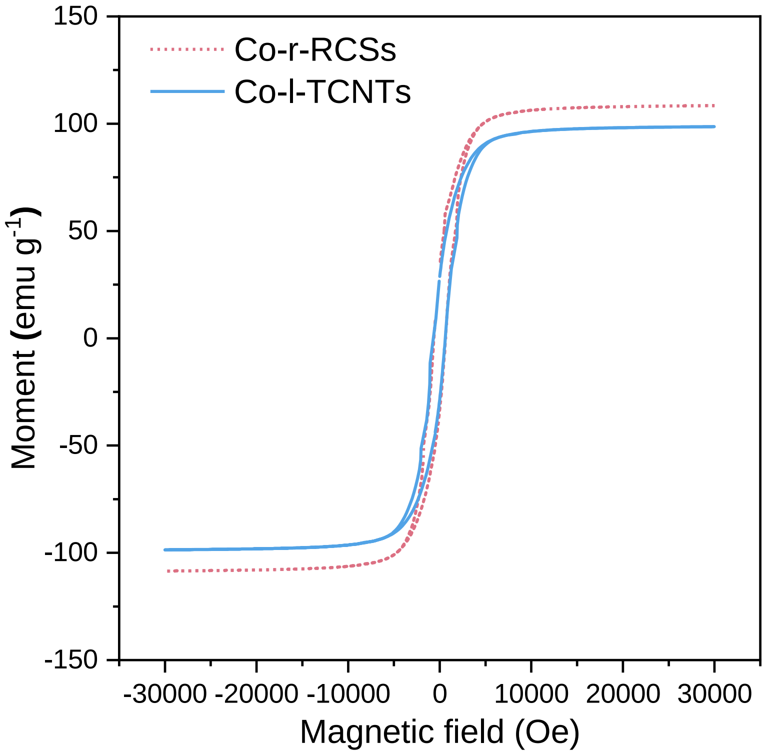

Figure S14. MH curves of Co-r-RCSs and Co-l-TCNTs samples.

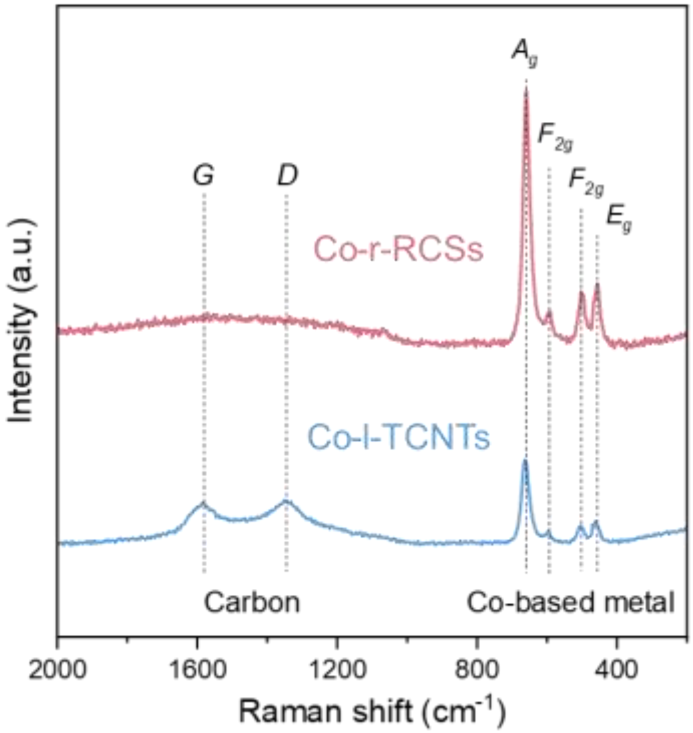

Figure S15. Raman spectra of Co-r-RCSs and Co-l-TCNTs.

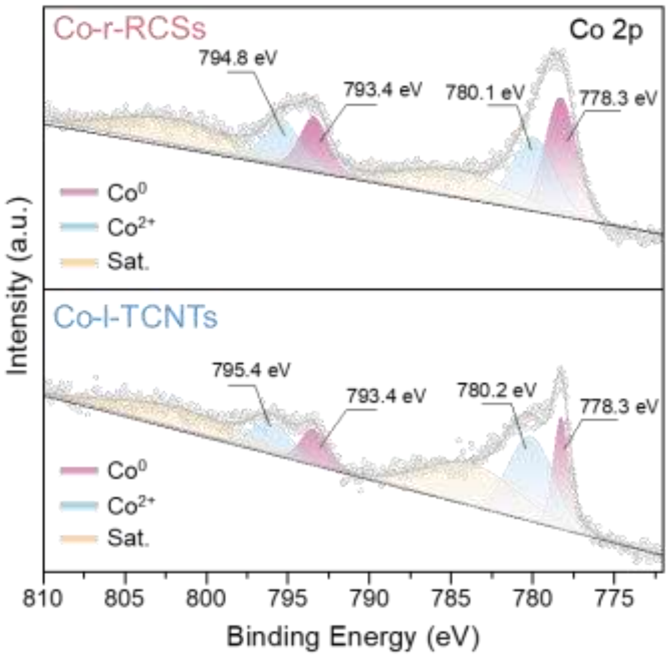

Figure S16. Co 2p XPS spectra of Co-r-RCSs and Co-l-TCNTs.

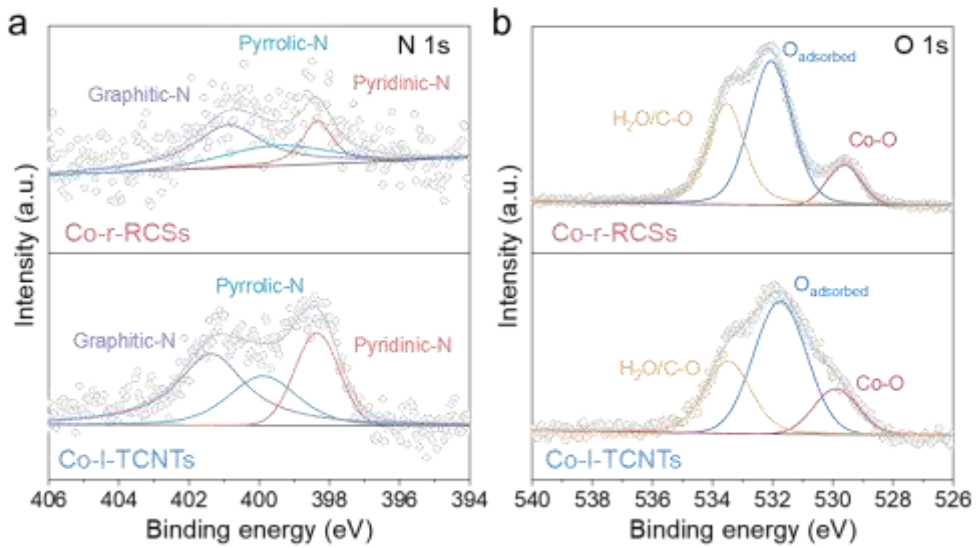

Figure S17. (a) N 1s and (b) O 1s XPS spectra of Co-r-RCSs and Co-l-TCNTs samples.

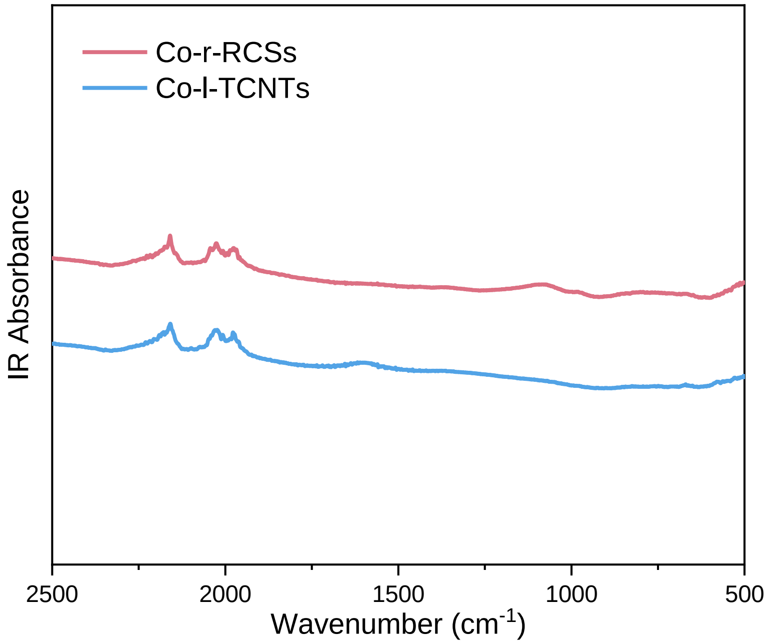

Figure S18. FTIR of Co-r-RCSs and Co-l-TCNTs samples.

FTIR tests show that there are no obvious organic functional groups in the two samples except for the environmental CO_2 peak (~2000 cm^{-1}).

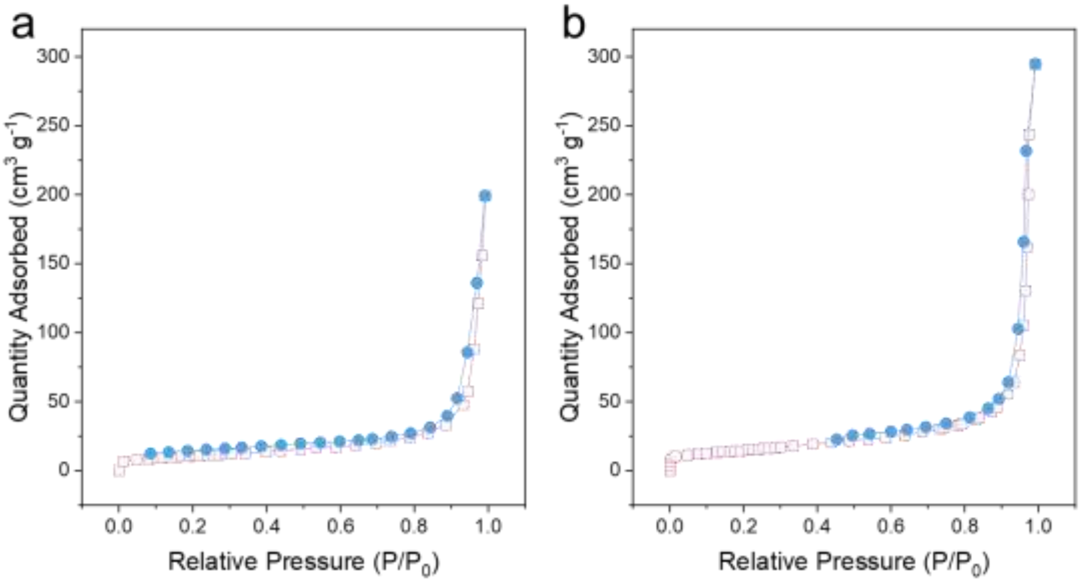

Figure S19. BET profiles of (a) Co-r-RCSs and (b) Co-l-TCNTs samples.

BET analysis shows that Co-r-RCSs and Co-l-TCNTs possess considerable specific surface area with typical type-IV isotherm characteristics.

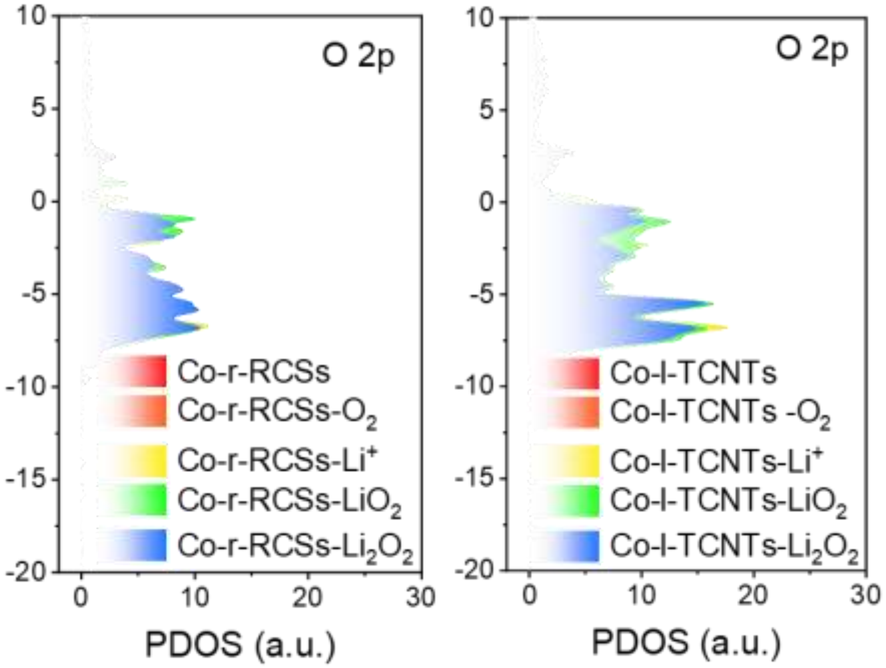

Figure S20. Computed PDOS of Co-r-RCSs and Co-l-TCNTs before and after adsorption of different species.

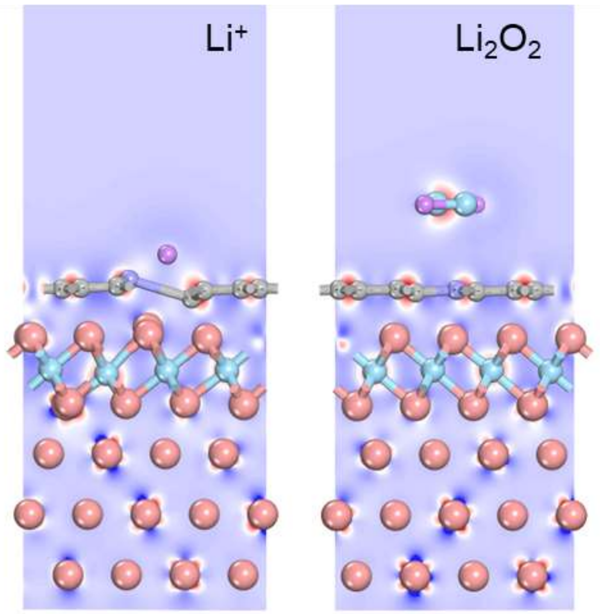

Figure S21. Charge density difference plots of Li^+ and Li_2O_2 on (111) surface for Co-r-RCSs electrode.

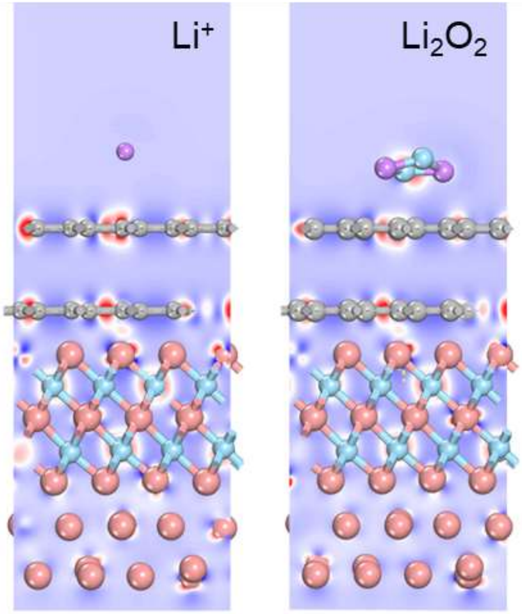

Figure S22. Charge density difference plots of Li^+ and Li_2O_2 on (111) surface for Co-l-TCNTs electrode.

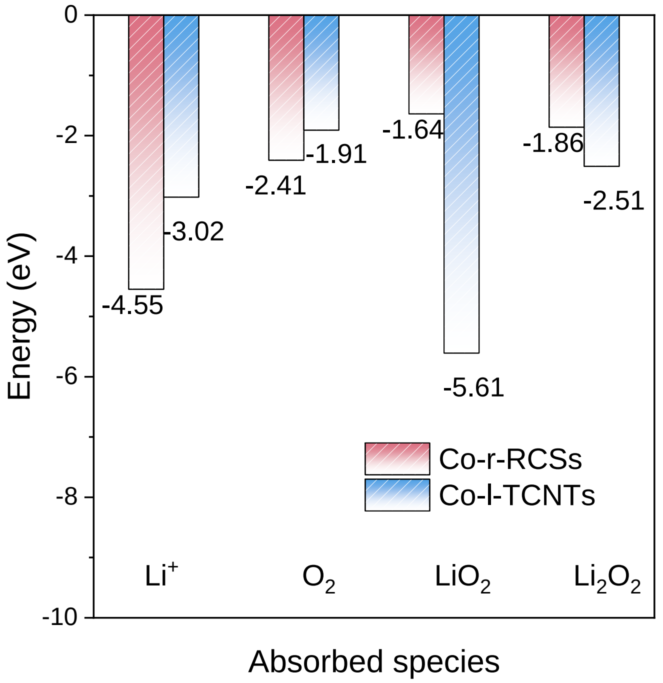

Figure S23. The binding energy of different adsorbates on Co-r-RCSs and Co-l-TCNTs electrodes.

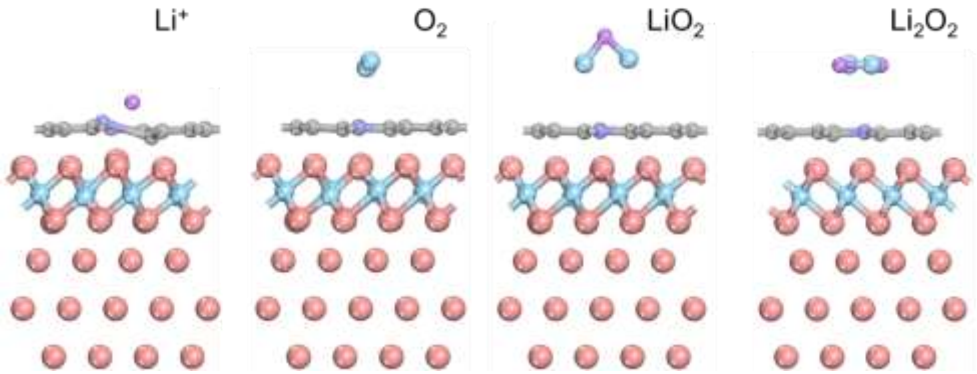

Figure S24. Optimized structure of different adsorbates on (111) surface for Co-r-RCSs electrode.

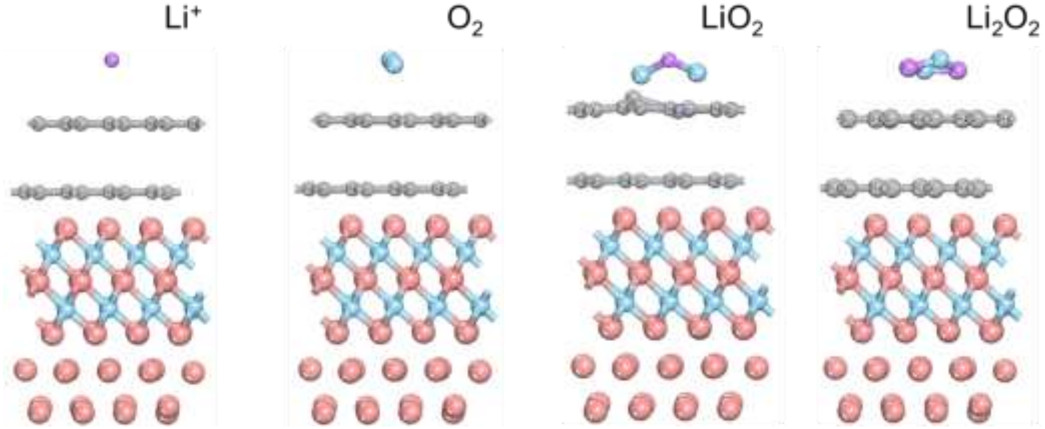

Figure S25. Optimized structure of different adsorbates on (111) surface for Co-l-TCNTs electrode.

The calculated electrochemical surface area (ECSA) of three electrodes: The ECSA of the three electrodes was calculated according to the CV curves. The following equations were used to calculate ECSA:[6,7]

$$C_T=\frac{I}{\left(\frac{d_E}{d_t}\right)} \tag{S1}$$

$$ECSA=\frac{C_T}{C^{*}\times A} \tag{S2}$$

where C_T is the total capacitance (F), I is the current (A), d_E/d_t is the voltage scan rate (V s^{-1}), C* is the specific capacitance, which can be obtained from the integration area of CV curves, A is the geometric area of the sample (cm^2). The calculated ECSA value of Co-r-RCSs electrode (9.65 cm^2_{ECSA}) is larger than that of Co-l-TCNTs (8.58 cm^2_{ECSA}) and Super P (4.64 cm^2_{ECSA}), which further proves that Co-r-RCSs electrode exhibits higher electrocatalytic activity.

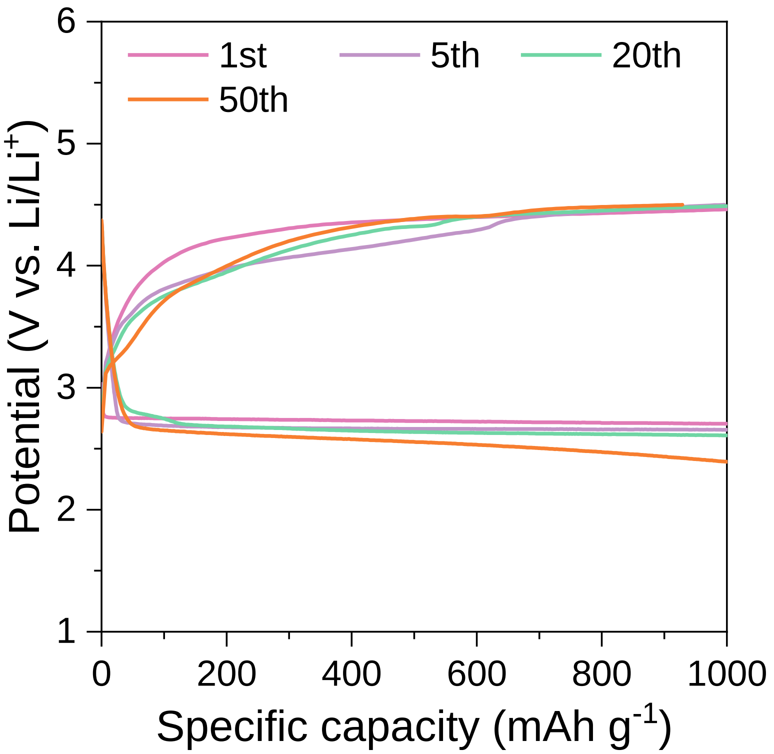

Figure S26. The discharge-charge curves of Super P electrode at a limited capacity of 1000 mAh g^{-1} and a current density of 400 mA g^{-1}.

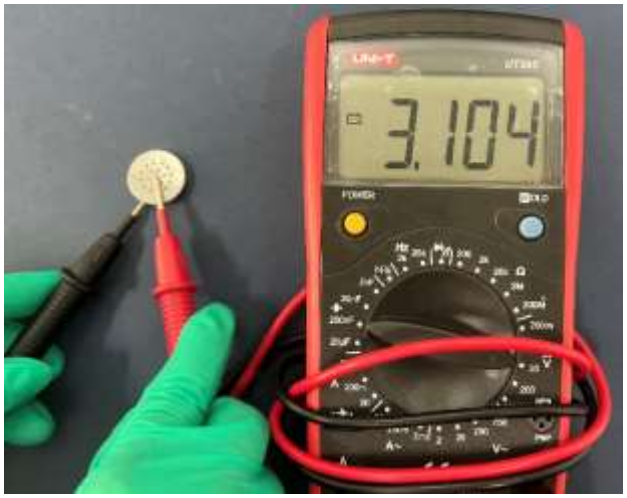

Figure S27. The open circuit voltage of button LOB with Co-r-RCSs electrode.

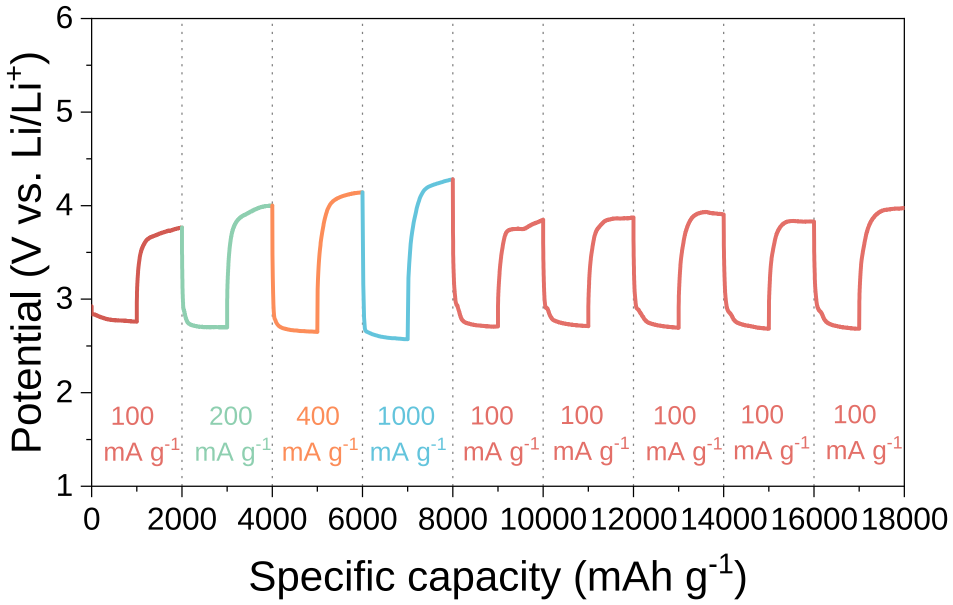

Figure S28. The discharge-charge profiles of Co-r-RCSs electrode at various current densities ranging from 0.1 to 1 A g^{-1} with a cut-off capacity of 1000 mAh g^{-1}.

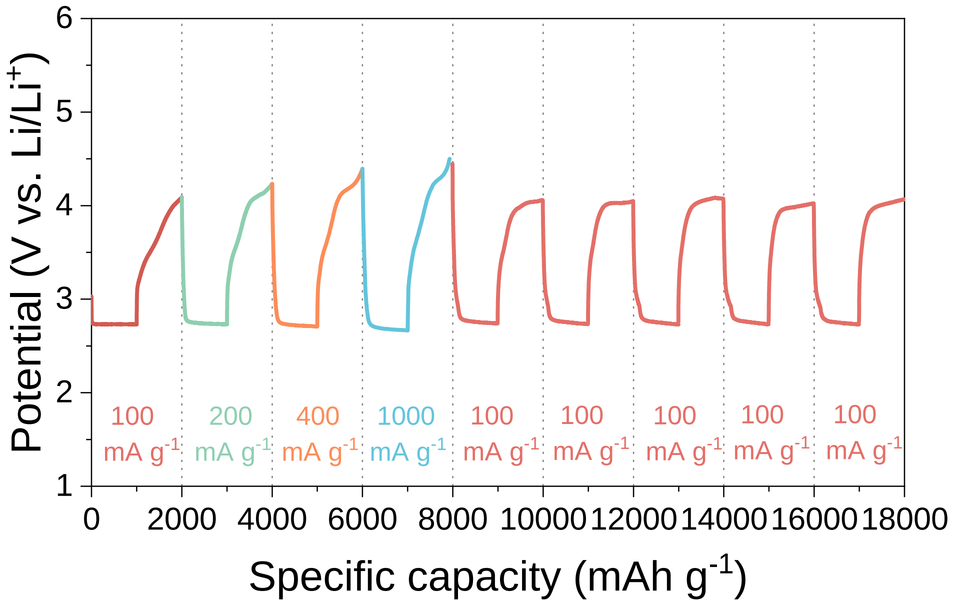

Figure S29. The discharge-charge profiles of Co-l-TCNTs electrode at various current densities ranging from 0.1 to 1 A g^{-1} with a cut-off capacity of 1000 mAh g^{-1}.

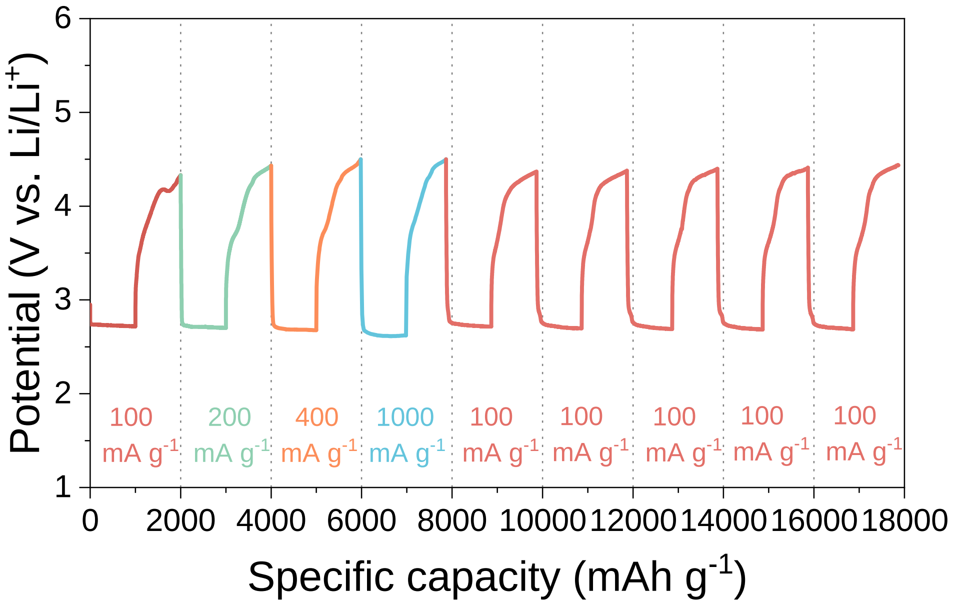

Figure S30. The discharge-charge profiles of Super P electrode at various current densities ranging from 0.1 to 1 A g^{-1} with a cut-off capacity of 1000 mAh g^{-1}.

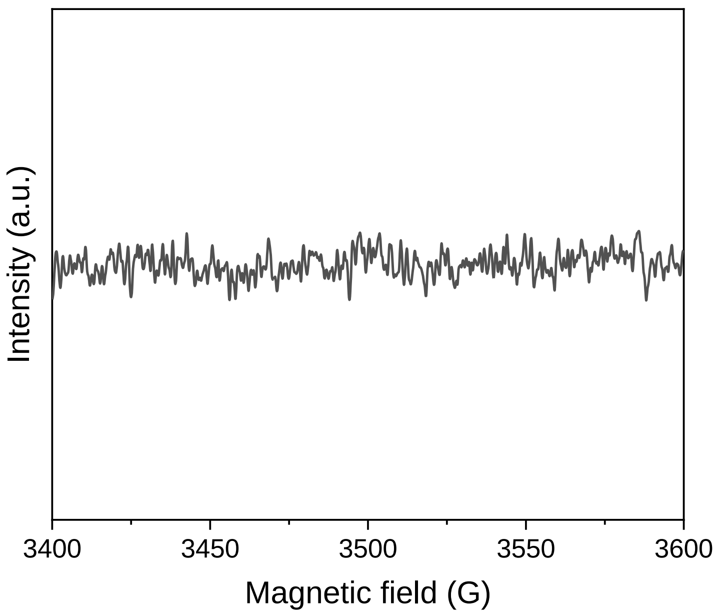

Figure S31. Ex-situ EPR spectra of the pristine separator.

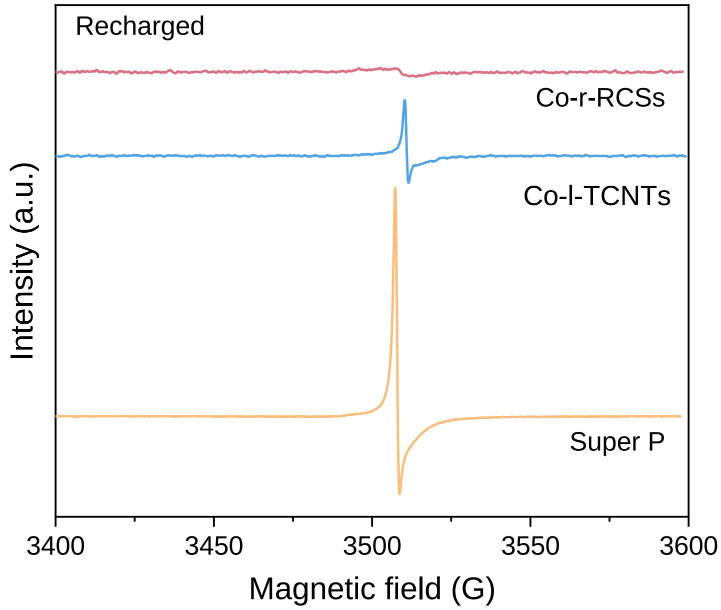

Figure S32. Ex-situ EPR spectra of the separators extracted from the recharged coin batteries with various electrodes.

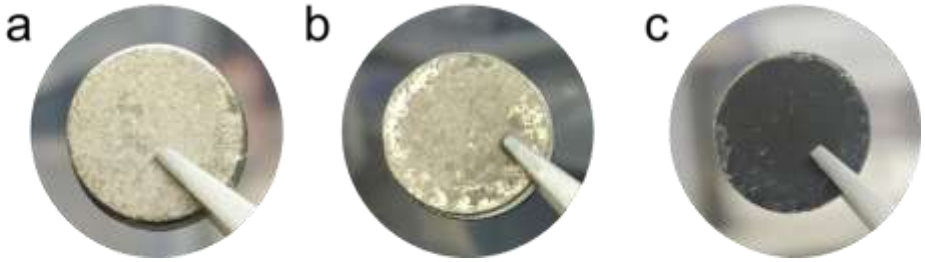

Figure S33. The recharged Li electrode of LOBs with (a) Co-r-RCSs, (b) Co-l-TCNTs and (c) Super P electrodes.

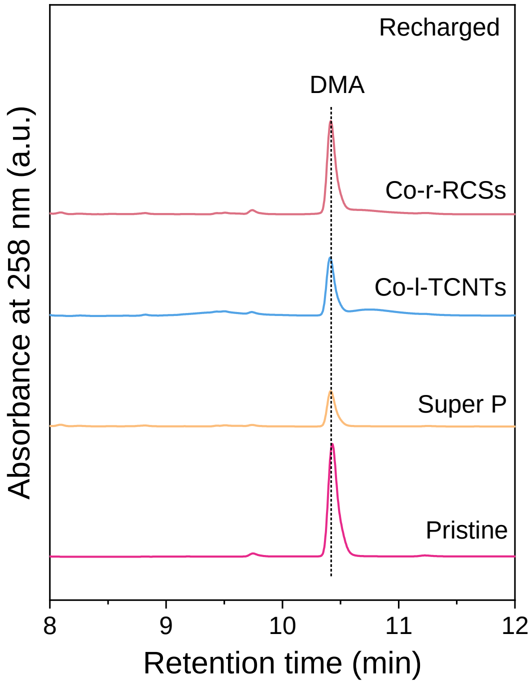

Figure S34. HPLC analyses of initial electrolyte and the electrolyte with different electrodes after 1st recharging cycle.

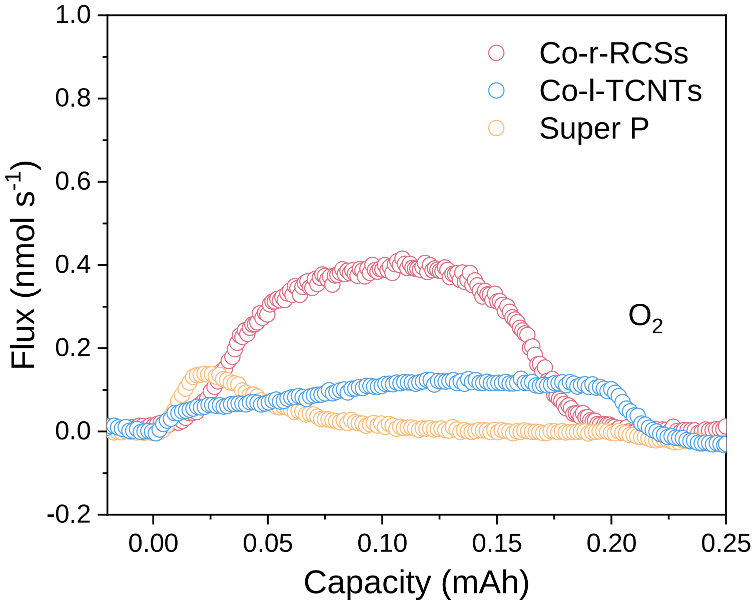

Figure S35. O_2 evolutions of LOBs with different electrodes using DMA-added electrolyte.

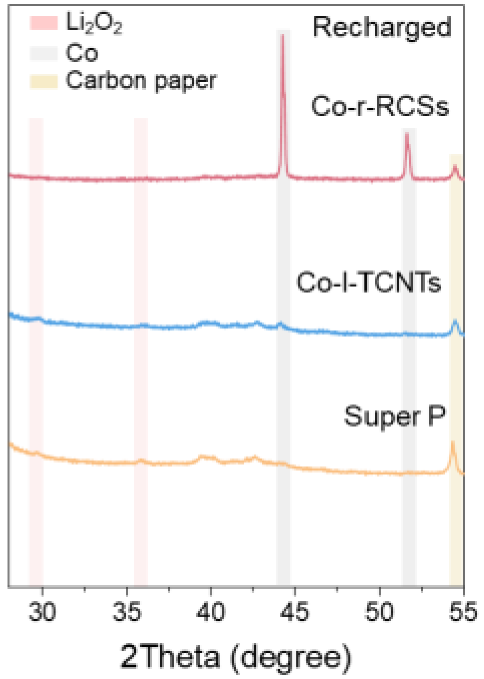

Figure S36. Ex-situ XRD patterns of various electrodes after 1st recharging cycle.

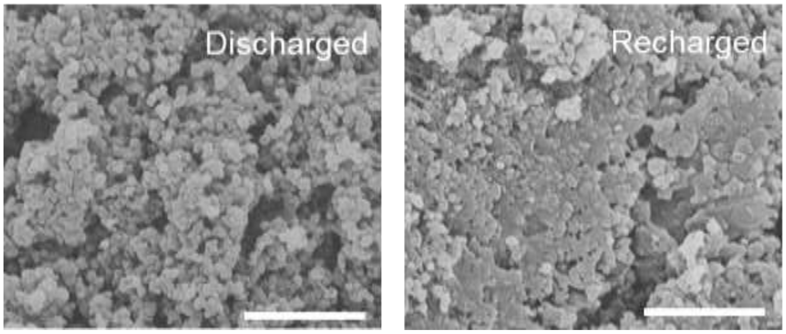

Figure S37. SEM of Super P electrode after 1st discharging/recharging. Scale bar: 1 μm.

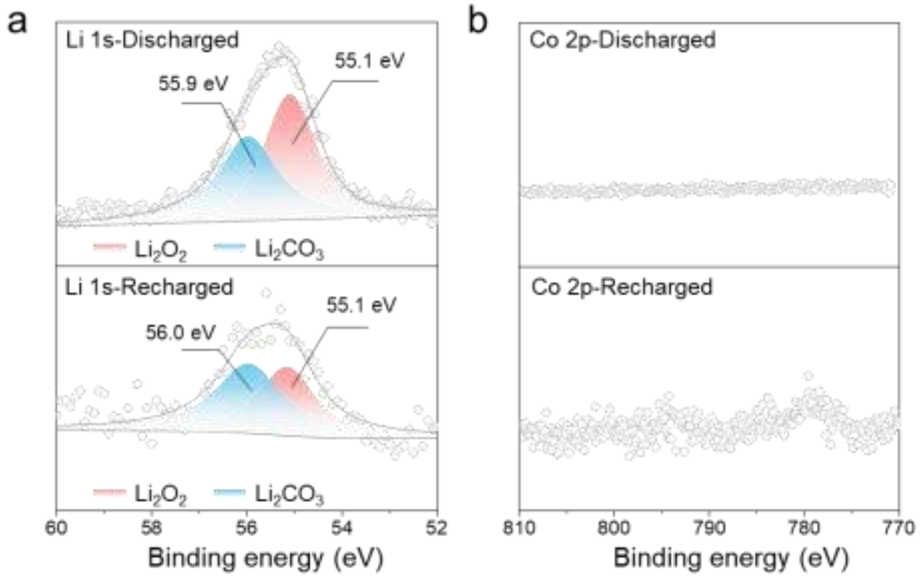

Figure S38. (a) Li 1s and (b) Co 2p XPS fitting of the Co-l-TCNTs electrode after 1st discharging and recharging.

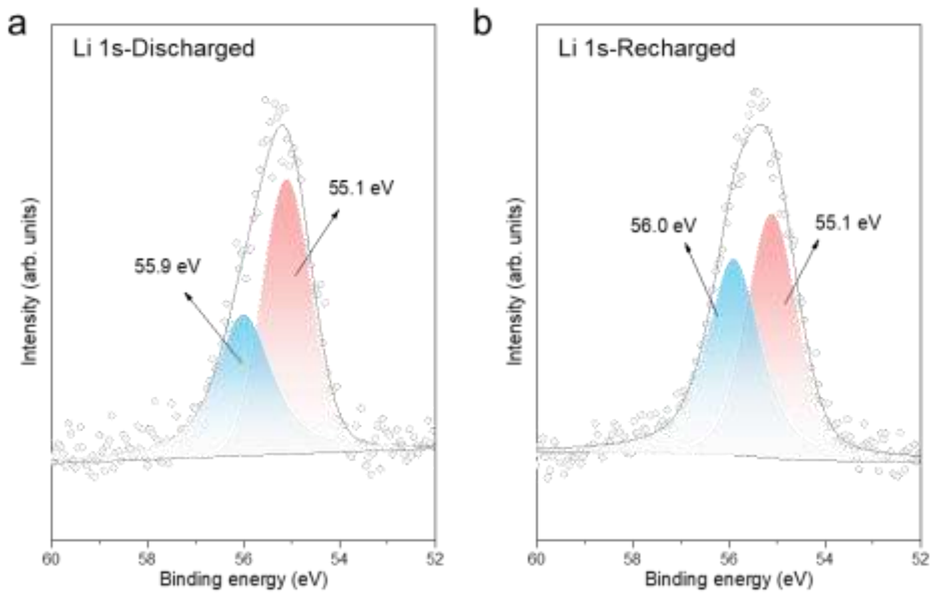

Figure S39. Li 1s XPS fitting of the Super P electrode after 1st (a) discharging and (b) recharging.

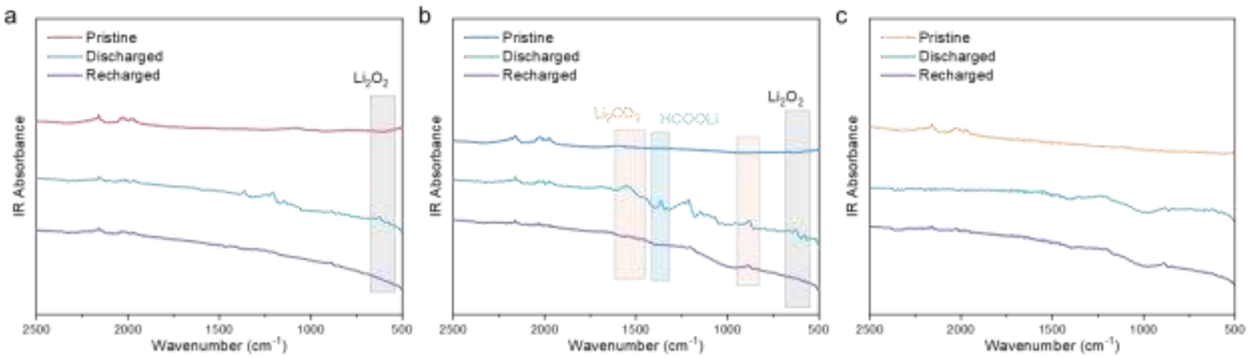

Figure S40. FTIR spectra of the (a) Co-r-RCSs, (b) Co-l-TCNTs and (c) Super P electrodes after discharging and recharging.

FTIR characterizations also confirm lower side product accumulations are generated over cycles in Co-r-RCSs (**Figure S40**a), further indicating its remarkable catalytic activity in boosting ORR/OER reversibility. In contrast, the cycled Co-l-TCNTs contains Li_2CO_3 (880, 1450, 1540 cm^{-1}), HCOOLi (1360 cm^{-1}) and Li_2O_2 (576, 625 cm^{-1}) (**Figure S40**b),[8] and slight Li_2O_2 was formed on the Super P (**Figure S40**c).

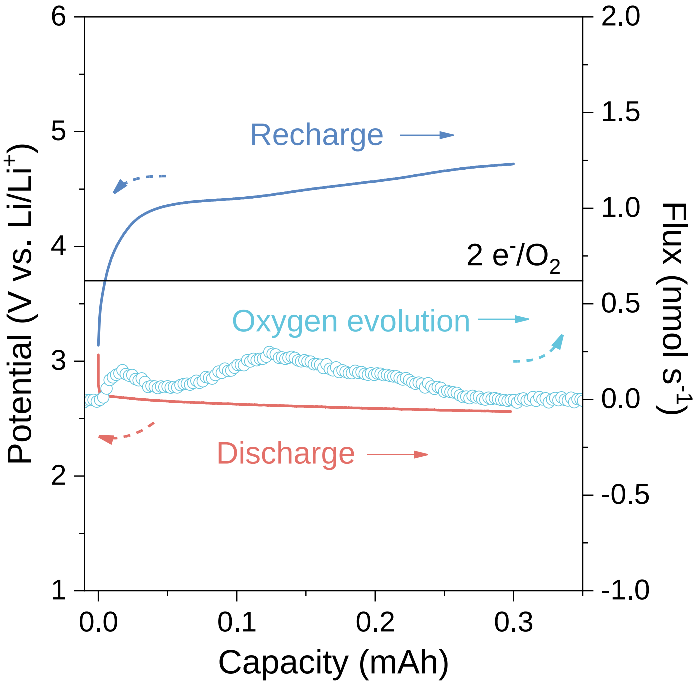

Figure S41. In-situ DEMS test of the Super P electrode during charge process.

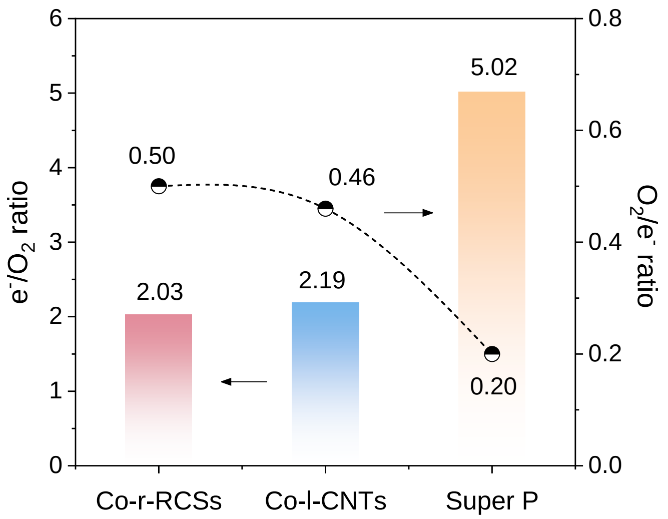

Figure S42. The ratio of e^-/O_2 and O_2/e^- of three electrodes during charge process.

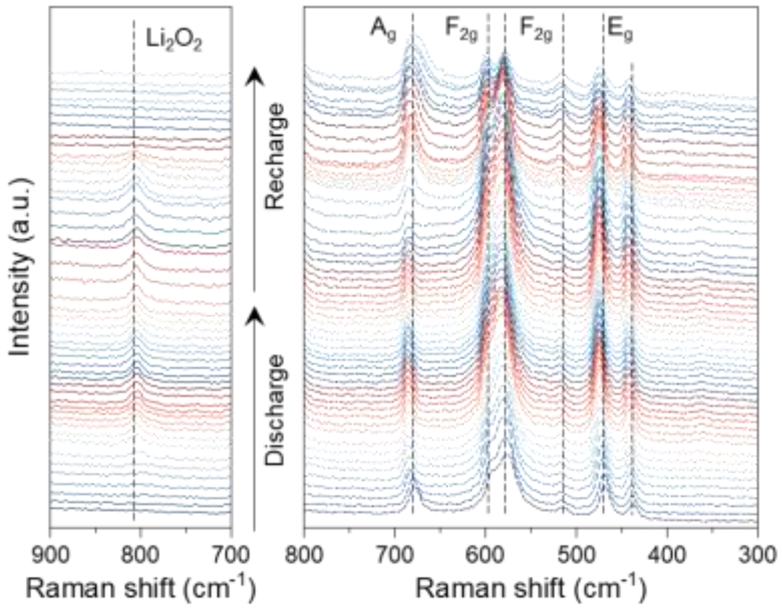

Figure S43. In-situ Raman contour diagram or spectra of Co-r-RCSs electrode at a current density of 400 mA g^{-1} with a fixed capacity of 1000 mAh g^{-1}.

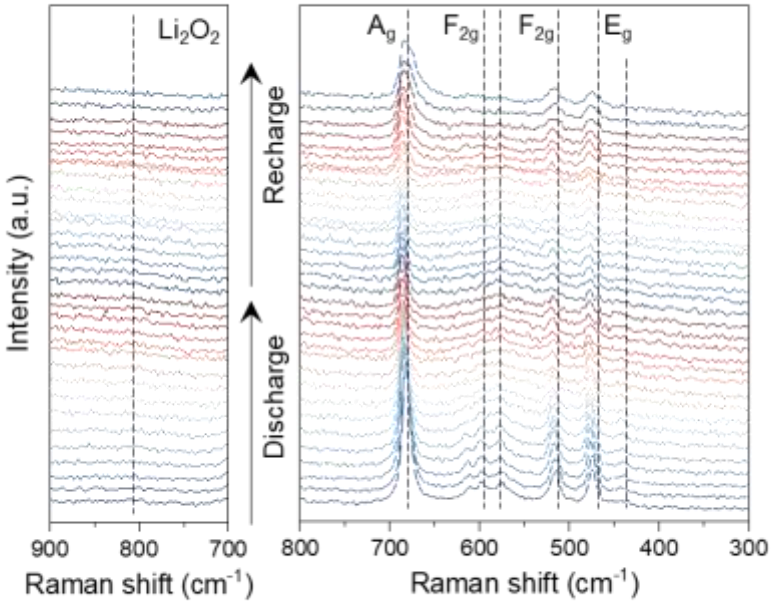

Figure S44. In-situ Raman contour diagram or spectra of Co-l-TCNTs electrode at a current density of 400 mA g^{-1} with a fixed capacity of 1000 mAh g^{-1}.

Table S1. Elemental distributions of Co-r-RCSs sample from TEM EDS-mapping.

Elements	wt%	Line type	K factor
C	57.24	K	2.782
N	0	K	3.532
O	3.53	K	2.029
Co	39.23	K	1.173

Table S2. Elemental distributions of Co-l-TCNTs sample from TEM EDS-mapping.

Elements	wt%	Line type	K factor
C	69.42	K	2.782
N	0	K	3.532
O	4.59	K	2.029
Co	25.99	K	1.173

Table S3. PDOS computing results of Co-r-RCSs catalyst before and after adsorption of different species.

Catalysts	Co 3d center	O 2p center	Co 3d-O 2p
Co-r-RCSs	-1.73123	-2.26563	0.53440
Co-r-RCSs-O_2	-1.69815	-2.31111	0.61296
Co-r-RCSs-Li^+	-1.59494	-2.33658	0.74164
Co-r-RCSs-LiO_2	-1.62714	-2.31111	0.68397
Co-r-RCSs-Li_2O_2	-1.60601	-2.31908	0.71307

Table S4. PDOS computing results of Co-l-TCNTs catalyst before and after adsorption of different species.

Catalysts	Co 3d center	O 2p center	Co 3d-O 2p
Co-l-TCNTs	-1.76044	-2.39170	0.63126
Co-l-TCNTs-O_2	-1.79630	-2.72479	0.92849
Co-l-TCNTs-Li^+	-1.76863	-2.39303	0.62440
Co-l-TCNTs-LiO_2	-1.78287	-2.39303	0.61016
Co-l-TCNTs-Li_2O_2	-1.79427	-2.63926	0.84499

References

1. Lin Y, Yang Q, Geng F *et al.* Suppressing singlet oxygen formation during the charge process of Li-O_2 batteries with a Co_3O_4 solid catalyst revealed by operando electron paramagnetic resonance. *J Phys Chem Lett* 2021; **12**: 10346-52.
2. Mahne N, Schafzahl B, Leypold C *et al.* Singlet oxygen generation as a major cause for parasitic reactions during cycling of aprotic lithium-oxygen batteries. *Nat Energy* 2017; **2**: 17036.
3. Clark SJ, Segall MD, Pickard CJ *et al.* First principles methods using CASTEP. *Z Krist-cryst Mater* 2005; **220**: 567-70.
4. Perdew JP, Burke K, Ernzerhof M. Generalized gradient approximation made simple. *Phys Rev Lett* 1996; **77**: 3865-8.
5. Park KW, Kolpak AM. Understanding photocatalytic overall water splitting on CoO nanoparticles: Effects of facets, surface stoichiometry, and the CoO/water interface. *Journal of Catalysis* 2018; **365**: 115-24.
6. Franz S, Arab H, Chiarello GL *et al.* Singl-step preparation of large area TiO_2 photoelectrodes for water splitting. *Adv Energy Mater* 2020; **10**: 2000652.
7. Huo J, Jin L, Chen C *et al.* Improving the sulfurophobicity of the NiS-doping CoS electrocatalyst boosts the low-energy-consumption sulfide oxidation reaction process. *ACS Appl Mater Inter* 2023; **15**: 43976-84.
8. Xiong Q, Huang G, Yu Y *et al.* Soluble and perfluorinated polyelectrolyte for safe and high-performance Li-O_2 batteries. *Angew Chem Int Ed* 2022; **61**: e202116635.
